# The world-wide waste web

**DOI:** 10.1038/s41467-022-28810-x

**Published:** 2022-03-29

**Authors:** Johann H. Martínez, Sergi Romero, José J. Ramasco, Ernesto Estrada

**Affiliations:** 1 Instituto de Física Interdisciplinar y Sistemas Complejos IFISC (CSIC-UIB), 07122 Palma de Mallorca, Spain; 2 Department of Biomedical Engineering, Universidad de los Andes, Cr 1 18A-12 Bogotá, Colombia; 3 Institute of Mathematics and Applications, University of Zaragoza, Pedro Cerbuna 12, Zaragoza, 50009 Spain; 4 ARAID Foundation, Government of Aragon, Zaragoza, Spain

**Keywords:** Environmental monitoring, Environmental monitoring, Applied mathematics

## Abstract

Countries globally trade with tons of waste materials every year, some of which are highly hazardous. This trade admits a network representation of the world-wide waste web, with countries as vertices and flows as directed weighted edges. Here we investigate the main properties of this network by tracking 108 categories of wastes interchanged in the period 2001–2019. Although, most of the hazardous waste was traded between developed nations, a disproportionate asymmetry existed in the flow from developed to developing countries. Using a dynamical model, we simulate how waste stress propagates through the network and affects the countries. We identify 28 countries with low Environmental Performance Index that are at high risk of waste congestion. Therefore, they are at threat of improper handling and disposal of hazardous waste. We find evidence of pollution by heavy metals, by volatile organic compounds and/or by persistent organic pollutants, which are used as chemical fingerprints, due to the improper handling of waste in several of these countries.

## Abstract (web summary)

The 2001–2019 web of international waste trade is investigated, allowing the identification of countries at threat of improper handling and disposal of waste. Chemical tracers are used to identify the environmental impact of waste in these countries.

## Introduction

Globally, 7–10 billion tonnes of waste are produced annually^1,2^, including 300–500 million tonnes of hazardous waste (HW)–explosive, flammable, toxic, corrosive, and of biological risk^3,4^. About 10%^5^ of this HW is traded through the *world-wide waste web* (W4). The W4 is a network formed by the legal trading of waste, where countries are represented as nodes and the flow of materials are encoded as weighted-directed links (edges). The forces that impulse international trade of HW are complex and multifactorial, and generally involve economic, geographic, socio-political, and environmental aspects difficult to disentangle. As a consequence, the volume of HW traded through the W4 in the last 30 years has grown by 500%^6^ and will continue to grow^7^, creating serious legal^8^, economic^6^, environmental^9^, and health^10^ problems at global scale.

It is frequently claimed that the global trade of HW is mainly a waste flow from developed to developing countries^5,11^. Although other studies point out a more complex picture with important contributions of the South–South trade^12^ as well as of South–North exports^13^.

From an economic perspective waste trade may offer benefits to both types of countries^9^. Developed countries would benefit from cheaper disposal costs in developing nations and avoiding increasing resistance to HW disposal facilities in their territories. Developing countries would gain access to cheap raw materials by recycling wastes, rocketing production, and employment. This would be a win-win situation if it were not because many of the importer nations are highly indebted countries with very poor track records of waste management and environmental performance^8^. In addition, as revealed by several high-profile cases^14^, the situation is aggravated by illegal HW trafficking to, and dumping in, developing countries^15^.

To address the problems of HW, the United Nations created in 1989 the Basel Convention (BaC) on the Control of Transboundary Movement of Hazardous Wastes and their Disposal^16^. The Convention has as mandate the monitoring of global waste trading. Countries self-report the amount of imported and exported waste, and its origin/destination. Some type of waste as radioactive materials are excluded from the reports. In its more than 30 years, BaC has revealed the difficulties to obtain accurate information regarding the magnitude and direction of global HW flows^5,17^. The information recorded by the BaC on waste trade does not contain information on illegal trade. However, it constitutes the most reliable information for building a map of the W4, which is vital to understand how the flows of HW are organized at global and local scales. This analysis is necessary for efficiently managing the transboundary HW trade and implementing more effective measures for its better management and control.

Here we rely on data reported by countries and territories on their trade of 108 categories of HW during the years 2001–2019, except for 2010, for which data is not available. This data is the most complete information about transboundary waste trade at the BaC database. By merging these categories into seven classes of waste, we study the trade networks that account for the legal flow of HW in the world. First, we analyze the global characteristics of these networks. By considering the relation between the simulated risk of waste congestion and countries’ environmental performance, we analyze the potential risks of improper handling and disposal of HW by individual nations. Finally, we identify “chemical fingerprints” that reveal the impact of improper handling and disposal of HW on the environment and human health in 28 countries identified at high risk.

## Results

### Theoretical modeling approach

We consider that the change of the amount of waste *w*_*i*_ in a country *i* over time obeys a logistic model:

1 $$\frac{d{w}_{i}\left(t\right)}{dt}={\dot{w}}_{i}\left(t\right)=\beta \left(1-{w}_{i}\left(t\right)\right)\mathop{\sum }\limits_{j=1}^{n}{A}_{ij}{w}_{j}\left(t\right),$$

where *β* quantifies how quickly waste grows per amount of waste already in the country and *A*_*ij*_ is the normalized amount of waste exported by country *i* to country *j*. In a period of time, let say from January to December of a given year, the amount of waste accumulated at a given country growth exponentially at earlier times if there is little waste at the beginning and enough processing resources in that country (including its exports of waste). But when the amount of waste gets large enough, processing resources start to get congested, slowing down the growth rate. Eventually, it will level off, completing a characteristic S-shaped curve. The amount of waste at which it levels off, represents the maximum amount of waste that this particular country can support, and it is called its carrying capacity. We say that a country which has reached its carrying capacity is congested or saturated. We consider here that a country *i* in the year *t* has a carrying capacity equal to the total amount of waste traded by it, i.e., imported and exported, that year. We normalize this carrying capacity for every country such that it is equal to one. In this way, all the countries will congest when ${\lim}_{t\to \infty }{w}_{i}\left(t\right)=1$. Let us call congestion time ${t}_{s}\left(i\right)$ to the time at which ${w}_{i}\left({t}_{s}\left(i\right)\right)=1$. Some countries will reach this congestion time earlier than others. Suppose that a country reaches its carrying capacity in a simulation time equivalent to February in the real-time. Then, because this situation is physically implausible we consider that such country is at risk of getting over-congested of waste during the rest of the year. In other words, we consider the congestion time as a proxy of the risk at which a country is exposed by the international trade of waste.

The first time derivative ${\dot{w}}_{i}\left(t\right)$ in the logistic model accounts only for properties of this change in an infinitely small neighborhood of the considered time *t*. However, the change of the amount of waste in a given country at *t* depends on the changes of input of wastes on finite (or infinite) time interval of the past. This is known as nonlocality by time or dynamic memory^18,19^. That is, the saturation of waste by a given country in a year depends not only on what it trades this year, but also on the waste “accumulated” by trading in the past by this country. In order to account for this temporal nonlocality or memory of the process, we will replace here the first time derivative by a fractional one. We use the Caputo time-fractional derivative, which when applied to a given function $f\left(t\right)$ is defined as^20^

2 $${D}_{t}^{\alpha }f\left(t\right):=\frac{1}{\varGamma \left(\kappa -\alpha \right)}\int\nolimits_{0}^{t}\frac{{f}^{\left(\kappa \right)}\left(\tau \right)d\tau }{{\left(t-\tau \right)}^{\alpha +1-\kappa }},$$

where *κ* = ⌈*α*⌉ is the ceiling function applied to *α*. Here, we consider 0 < *α* ≤ 1 and *κ* = 1.

In order to understand how the Caputo fractional derivative captures the past history of the system^21^, let us write it as

3 $${}^{C}{D}_{t}^{\alpha }=\frac{1}{\varGamma (1-\alpha )}{\int _{0}^{t}}\left[\frac{1}{{(t-s)}^{\alpha }}\right]\frac{df(s)}{ds}.$$

The term in the squared parenthesis inside the integral represents a weight given to the standard derivative. Then, we can consider the initial time of the integration as the remote past, while *t* is the present. Therefore, when we consider a time *k* far apart from the present *t*, the term *t* − *s* is relatively large, thus if *α* = 1 the term (*t* − *s*)^−*α*^ vanishes, which indicates no contribution from this remote past time. However, if α ≪ 1 such term is different from zero and the derivative $\frac{df(s)}{ds}$ receives a weight different from zero for the contribution of this remote past time. In closing, the smaller the value of *α* the larger the contribution from the remote past^21^. That is, the system remembers its past history. If *α* = 1, only the present time is considered by the derivative, which indicates no memory from the past history of the system.

The waste congestion of a given country can be produced either (i) because it imports large amounts of waste from other countries, or (ii) because it produces large amounts of waste which it cannot process with its infrastructures. The first could be for instance the case of China before 2017, where an estimate of 70% of the world’s e-waste ends up in Guiyu, in Guangdong Province where no more than 25% is recycled in formal recycling centers. The second can be exemplified by the case of household waste in Senegal, where the lack of infrastructures and collection system makes the problem insurmountable by local authorities. Senegal exported more than 15,000 tonnes of household waste to Italy in 2009. To differentiate both situations we will designate them as (i) congestion at arrival, for the case where congestion can be produced by importing large amounts of a given type of waste; and (ii) congestion at departure, for the case where congestion can be produced due to the existence of large amounts of waste in a country, which are then exported to another. Also, we should notice here that the amount of waste of a given type reported by a country A as exported to a country B is not always equal to the amount of the same waste reported by B as imported from A. This difference could be due to several causes which escape the analysis of the current work, but the split of congestion at arrival and at departure avoids any problem arising from this data asymmetry.

We then follow Lee et al.^22^ and make a change of variable in the model: ${s}_{i}\left(t\right):=-\log \left(1-{w}_{i}\left(t\right)\right)$, such that ${s}_{i}\left(t\right)$ represents the “information content” that country *i* is not congested at time *t*. Using this approach and considering ${w}_{i}\left(0\right)={w}_{0}$ as an initial condition, we define here the following models of waste congestion in the W4 with dynamic memory:

(i) Congestion at arrival

4 $${D}_{t}^{\alpha }\,{{{{{{{{\bf{s}}}}}}}}}_{A}(t)={\beta }_{A}^{\alpha }\,A\,{{{{{{{\bf{w}}}}}}}}\left(t\right),$$

(ii) Congestion at departure

5 $${D}_{t}^{\alpha }\,{{{{{{{{\bf{s}}}}}}}}}_{D}(t)={\beta }_{D}^{\alpha }\,{A}^{T}\,{{{{{{{\bf{w}}}}}}}}\left(t\right),$$

where *A*^*T*^ is the transpose of the weighted adjacency matrix *A* of the network, and **s**_*A*_, **s**_*D*_, and **w** are vectors formed by an element of *s* and *w* per node. Due to the uncertainties in the parameters involved in these equations we consider here a “worst-case-scenario” approach in solving them. That is, instead of obtaining the solution of these equations we will solve a linearized version of them, whose solution is an upper bound to the exact solution^23^:

6 $${D}_{t}^{\alpha }{\hat{{{{{{{{\bf{s}}}}}}}}}}_{\ell }(t)={\beta }_{\ell }^{\alpha }\,\hat{B}\,{\hat{{{{{{{{\bf{s}}}}}}}}}}_{\ell }(t)+{\beta }_{\ell }^{\alpha }\,B\,{{{{{{{\bf{b}}}}}}}}\left({{{{{{{{\bf{w}}}}}}}}}_{{{{{{{{\bf{0}}}}}}}}}\right),$$

where ${{{{{{{\bf{b}}}}}}}}\left({{{{{{{{\bf{w}}}}}}}}}_{0}\right):={{{{{{{{\bf{w}}}}}}}}}_{0}+\left({{{{{{{\bf{1}}}}}}}}-{{{{{{{{\bf{w}}}}}}}}}_{0}\right)\log \left({{{{{{{\bf{1}}}}}}}}-{{{{{{{{\bf{w}}}}}}}}}_{0}\right)$ with the logarithm taken entrywise, *ℓ* = {*A*, *D*}, $\hat{B}=B\,{{\Omega }}$, ${{\Omega }}=\,{{\mbox{diag}}}\,\left({{{{{{{\bf{1}}}}}}}}-{{{{{{{{\bf{w}}}}}}}}}_{0}\right)$ (**1** is an all-ones vector), and $B=\left\{A,{A}^{T}\right\}$. The solutions of these equations (expressed as normalized amounts of waste, ${\hat{{{{{{{{\bf{w}}}}}}}}}}_{\ell }\left(t\right)$) are nondivergent upper bounds to the exact solutions of the Eqs. (4) and (5), respectively. That is, ${\hat{{{{{{{{\bf{w}}}}}}}}}}_{\ell }\left(t\right)\succcurlyeq {{{{{{{{\bf{w}}}}}}}}}_{\ell }\left(t\right)$ where ≽ indicates that the inequality is obeyed for every entry of the vectors. Therefore, ${\hat{{{{{{{{\bf{w}}}}}}}}}}_{\ell }\left(t\right)$ represent the worse-case-scenario of congestion at arrival and at departure for every country in the W4. Let the initial condition be *γ* = *w*_0_ = 1 − *c*/*n* with *c* is a small number, i.e., *c* ≪ 1 (we took *c* = 0.005), and *n* is the number of nodes in the network. Then the solution of (6) is given by (see SI for details)^23^:

7 $${\hat{{{{{{{{\bf{s}}}}}}}}}}_{\ell }(t)=	 \left(\frac{1-\gamma }{\gamma }\right)\,{E}_{\alpha ,1}\left({t}^{\alpha }\,{\beta }_{\ell }^{\alpha }\,\gamma \,B\right)\,{{{{{{{\bf{1}}}}}}}}\\ 	-\,\left(\frac{1-\gamma }{\gamma }+\log \gamma \right){{{{{{{\bf{1}}}}}}}},$$

where ${E}_{\alpha ,1}\left({t}^{\alpha }\,{\beta }_{\ell }^{\alpha }\,\gamma \,B\right)$ is the Mittag-Leffler matrix function of *B*^24^.

The fact that the solution of (4) is given by means of Mittag-Leffler matrix functions implies that congestion parameters, e.g., congestion time, depends on the global interactions of countries in the network and cannot be derived from simple static (centrality) measures (see SI for explanation). This model estimates the time at which a country saturates of a given type of waste. From this value alone we cannot infer whether that country is at risk of improper handling and disposal of wastes. To illustrate this situation let us consider two countries which saturate of type I waste at the same time: Japan and Afghanistan. They reach their 50% of congestion at *t* = 28654.54 (risk of waste congestion is 0.606). However, while Japan is one of the richest countries in the world with GDP ranging 4.002–4.591 trillion USD, Afghanistan is among the poorest ones with GDP of 7.521–21.972 billions USD for the period of time considered here. This obviously gives these countries very different capacities for managing a waste congestion, a situation which is well reflected in the environmental track record of each of these countries. We will account for these differences in the section “Potential environmental impact”.

### Structural analysis of W4

During 2001–2019, the total amount of wastes reported by the BaC around the world was 1,470,096,618 metric tonnes (which is more than 4000 times the weight of the Empire State building). Time-aggregated weighted-directed networks of seven types of waste grouping together 108 BaC categories were created as described in “Methods”. The distribution of wastes by the different types considered here (see “Methods”) is very unequal with a large concentration on the wastes of types I–III. These three types of wastes account for 95.41% of the total weight of wastes traded in the period of study. We then focus here on these three types and the rest are considered in the SI. Waste of type I accounts for 40.4% of the total volume of wastes traded world-wide in the period of study, followed by type II (28.9%) and type III (26.1%).

For the period 2001–2019, most of the international trade of type I–III wastes took place between developed nations. They accounted for 90.67% (8.29 × 10^7^ tons of type I), 70.19% (4.58 × 10^7^ tons of type II), and 99.07% (5.86 × 10^7^ tons of type III) of the total volume of waste traded in that period.

**Fig. 1:**
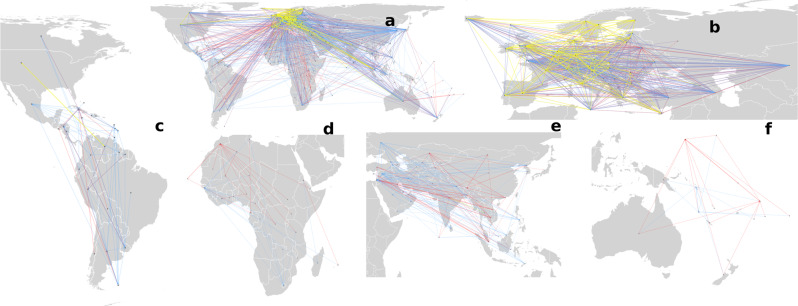
The world-wide waste web. Superposition of the W4 networks of types I (red edges), type II (blue edges), and type III (yellow edges) of waste, where the nodes represent the countries which traded the corresponding waste in the years 2001–2019. The direction of the edges indicates the flow from exporter to importer as reported in the BaC database. A view of the global network in (**a**), with zooms for the local networks of Europe in (**b**), the Americas (**c**), Africa (**d**), Asia (**e**), and Oceania (**f**). Map tiles by Bjorn Sandvik, under CC BY-SA 3.0 available at http://thematicmapping.org/downloads/world_borders.php.

A closer inspection of the W4 (see Fig. 1) reveals a large imbalance in the directionality of the HW trades between developed, developing, and least-developed countries. In the case of wastes of type I—which include clinical, medical, and pharmaceutical wastes as well as residues from industrial waste disposal operations—developed nations exported more to the developing and least-developed world than what they import from them, i.e., 4,340,000 and 25,500 tons, respectively. Even for the case of household wastes (type III) developed nations exported 52,000 and 15,300 tons more than what they imported from developing and least-developed nations, respectively. Only in the case of wastes of type II, which contains many valuable metals, the developed nations imported more than what they exported to developing nations, i.e., 9,870,000 tons. The exports and imports of types I–III display fat-tailed distributions, indicating the existence of a relatively small number of exporters/importers which concentrate most of the volume and number of connections in the W4.

### Potential environmental impact

**Fig. 2:**
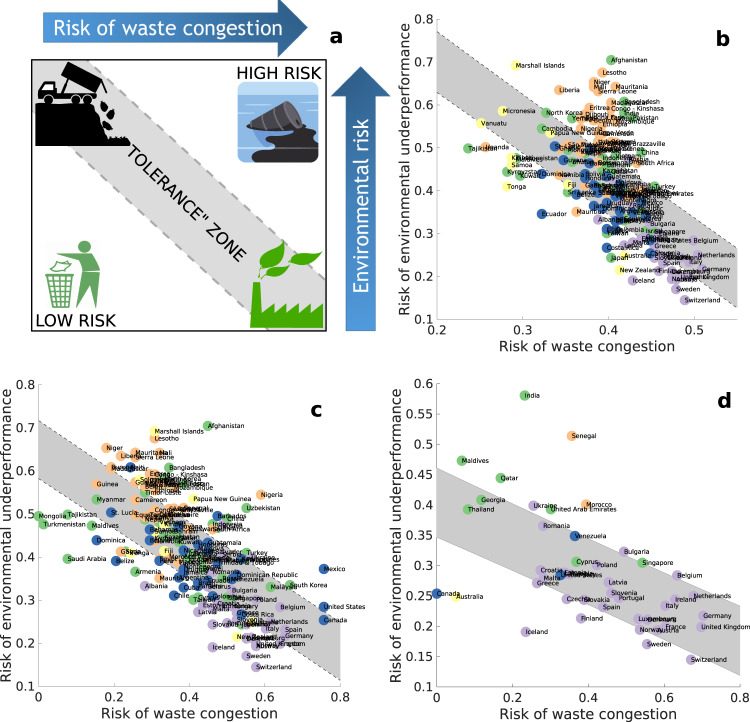
Potential environmental impact of waste congestion (PEIWC). Plot of the risk of waste congestion versus the environmental performance (**a**) indicating the central region of “tolerance” where countries process waste with relatively low environmental and human health impacts. The tolerance zone is defined here by the upper and lower 50% prediction bounds for response values associated with the linear regression trend between the two risk indices. Countries over the tolerance zone are at high risk of improper handling and disposal of wastes (HRIHDW). **b**–**d** Illustration of the PEIWC for wastes of types I–III, respectively. Nodes are colored by the continent to which the country belongs to: blue (Americas), purple (Europe), orange (Africa), green (Asia), yellow (Australia/Oceania). Icons of panel **a** were obtained from https://www.pdclipart.org/ under CC Public Domain.

As we have mentioned before, two countries with the same congestion time of a given type of waste may have very different capacities for processing it. These differences can be reflected in the environmental impacts that such waste has in the countries. In order to capture these differences, we use here the “Environmental Performance Index (EPI)” measured by a set of parameters as described in ref. ^25^. Returning to the example of Japan and Afghanistan, which saturate of waste type I at the same time, it should be noticed that the EPI of Japan of 75.6 contrasts with that of Afghanistan of 29.5. In our framework, we build networks by considering HW type and the processing capacities of the countries in each waste type. Then we calculate the corresponding risk that each waste poses before aggregating to a global score. Thus, we introduce the potential environmental impact of waste congestion (PEIWC) (see “Methods”). In Fig. 2a, we illustrate a typical PEIWC. Ideally, those countries with poor EPI should manage low volumes of HW. They should appear at the top-left corner of the PEIWC. Those countries with good EPI and low levels of HW congestion should appear in the low-right corner of the PEIWC. The central zone represents a “tolerance” zone, where countries manage wastes according to their capacities and their environmental responsibilities. However, there are countries with poor EPI that may congest very quickly. They are located over the tolerance zone and represent countries with high risk of improper handling and disposal of wastes (HRIHDW).

In Fig. 2b–d, we illustrate the PEIWC for wastes of types I–III. We identified 57 countries at HRIHDW: 29 from Africa, 16 from Asia, 5 from the Americas, 4 from Europe, and 3 from Oceania. The color codes in Fig. 2 clearly reveal the geographical distribution of countries at different levels of risk of improper handling and disposal of wastes.

At the left-bottom side of the PEIWC there are a variety of countries/territories of different sizes and levels of development, which are characterized by processing efficiently the waste they receive, independently of its amount. That is, these countries have developed capacities for processing the amounts of waste they receive without compromising the environment. This situation could be metastable and redirecting more waste to these countries without increasing their processing capacities could trigger waste-driven ecological problems. However, due to the environmental good track record of these countries they may represent opportunities for responsible investment in waste treatment technologies.

**Fig. 3:**
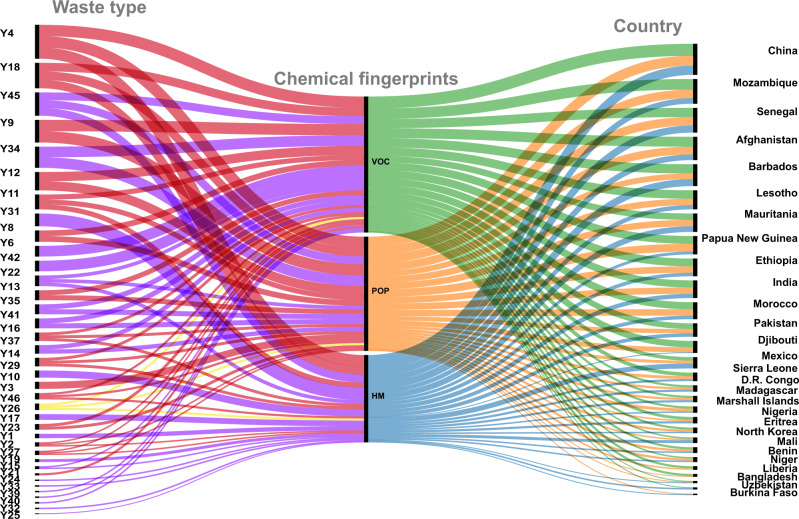
Chemical fingerprints of waste. The three classes of chemical fingerprints: Volatile Organic Compounds (VOC) (green), Persistent Organic Pollutants (POP) (orange), and HM (blue) left by the three BaC waste types Y1-Y18 (red), Y19-Y45 (purple), Y46-Y47 (yellow) in the top 28 countries at high risk of improper handling and disposal of wastes (HRIHDW).

It is difficult to find non-anecdotal evidence on the impact of waste trade on different countries in the W4. That is, if we want to move away from factual claims relying only on personal observations, collected in a casual or non-systematic manner about the impact of waste trade we should find some “markers” that can be traced quantitatively from waste to ecological impact. We propose here the use of Chemical Fingerprints (CF) as such markers for the analysis of the impact of waste trade on HRIHDW. A CF is a chemical or group of chemicals that are generated by wastes and leave a quantifiable trace on the environment. In the case of wastes of types I–III we have found that they may leave environmental and/or human health CF in the form of (i) heavy metals (HM)^26^, (ii) volatile organic compounds (VOC)^27,28^, and persistent organic pollutants (POP)^29^. In Fig. 3, we illustrate the connections between the different kinds of wastes, the chemical fingerprints present in them and some of the countries found here at HRIHDW (see further discussion for a detailed analysis).

**Fig. 4:**
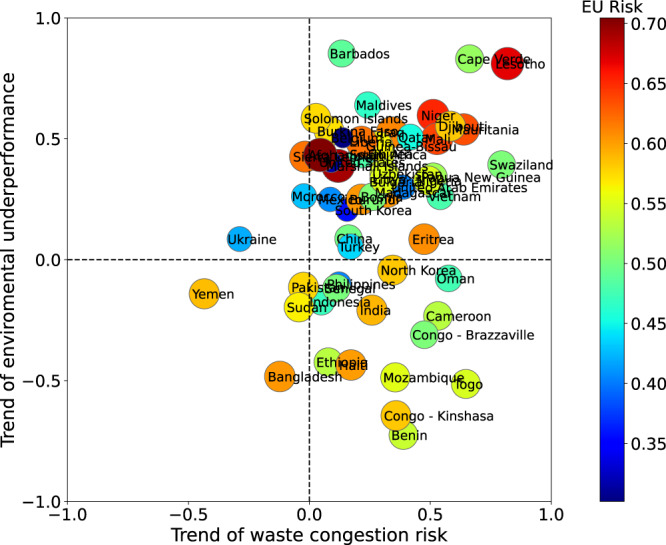
Temporal trend (period 2001–2019) of the waste congestion risk and of the environmental underperformance risk for some countries at HRIHDW. The trend is measured by the Pearson correlation coefficient between the corresponding variable and the years in the period. Bottom-left quarter identifies the countries with a trend to improve both indices. Top-right quarter identified those countries with a trend to deterioration of both indices. EU Risk stands for Environmental Underperformance Risk.

We also study waste-aggregated W4 networks for every year in the 2001–2019 period. Temporal trends of the waste congestion and environmental underperformance risks were built for 57 countries at HRIHDW (Fig. 4). What we plot here is the Pearson correlation coefficient of the waste congestion risk vs. time (x-axis) as well as of the EU vs. time (y-axis). Therefore, a negative value on the x-axis indicates that the corresponding country has dropped its waste congestion risk. Similarly, a negative value on the y-axis indicates that the country improved its environmental performance. Very few countries display a tendency to improve both risk indices (bottom-left quarter), while the majority showed simultaneous detriment of both (top-right quarter) from 2001 to 2019. The values plotted here should not be confused with those in Fig. 2, where we plotted the absolute risks of waste congestion and of environmental underperformance for countries. For instance, Lesotho and Bangladesh are both at HRIHDW for waste of types I and II, i.e., they are at the top-right corner of Fig. 2b and c. However, Lesotho is one of the countries which have worsened both, its waste congestion risk and its environmental performance in the period 2001–2019 (top-right corner of Fig. 4), while Bangladesh has significantly improved its environmental performance and slightly improved its risk of waste congestion (bottom-left corner of Fig. 4).

For a better characterization of the global structures of these 18 networks (one per year of the period analyzed), we calculated several of their topological features. The edge density of these networks displays a significant decreasing tendency along the period of time analyzed, i.e., the Pearson correlation coefficient with time is *r* = −0.69. The trade networks have become “smaller-worlds” from 2001 to 2019 with a positive trend of the average clustering coefficient (*r* = 0.59) and a negative one of the average path length (*r* = −0.36). The average reciprocity of edges between pairs of connected countries was relatively stable across the period (*r* = −0.05). We should notice that this index does not account for the weights of the edges. However, the average number of weighted triangles displayed a very strong negative tendency from 2001 to 2019 (*r* = −0.83), followed by a similar trend of the weighted subgraph centrality of the countries (*r* = −0.78). The implications of these findings for the international waste trade are analyzed in the next section.

## Discussion

Here we focus the discussion of the results found in the previous section on the analysis of CF in HRIHDW. For that purpose, we performed an intensive literature search that allowed to trace back some CF generated by the types of wastes that produce the high risk in those countries to their environmental and/or animal/human health effect. As mentioned before, in Fig. 3 we illustrate the connections between the BaC wastes Y1-Y47, their CFs and the top 28 countries at HRIHDW (see “Methods”).

### Heavy metals

Waste is one of the main anthropogenic sources of HM in the environment^26,30^, with electrical and electronic waste (e-waste) alone containing 56 metals^31^. We focus here on 8 HM ubiquitous in wastes of different kinds. Lead (Pb), cadmium (Cd), nickel (Ni), mercury (Hg), chromium (Cr), zinc (Zn), copper (Cu), and arsenic (As), appear in waste from pesticides, medicines, paints, dyes, catalysts, batteries, electronic devices, industrial sludge, printing products, incineration of household wastes, among others^26,30^ (see SI).

**Fig. 5:**
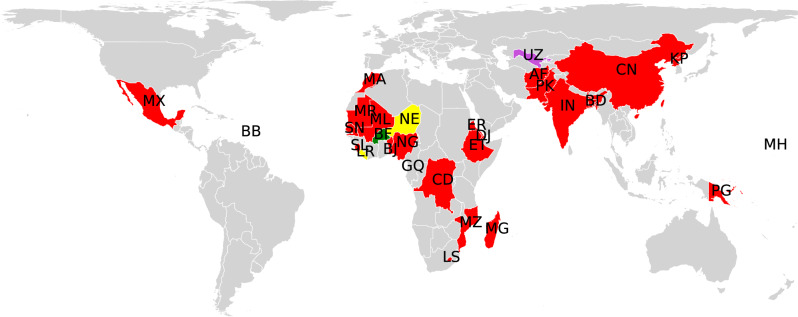
High risk of improper handling and disposal of wastes. Illustration of countries at HRIHDW of types I–III wastes and the chemical fingerprints left by these HW in their environment and/or human health. Countries with impact of heavy metals (HM) and persistent organic pollutants (POP) (green), volatile organic compounds (VOC) and HM (purple), VOC, HM and POP (red), VOC and POP (yellow) are illustrated. Map tiles by Bjorn Sandvik, under CC BY-SA 3.0 available at http://thematicmapping.org/downloads/world_borders.php.

In total, from the 28 countries at HRIHDW there are 24 ones in which waste traded through the W4 can leave CF in the form of HM (see Fig. 5). We have found that the major sources of waste-generated HM are the open (unregulated) dumpsites existing in many developing countries as well as the (informal) recycling of wastes, principally of e-waste. To have a clearer picture of the situation we refer to the data of the period 1990–2015 quantifying the amount of hazardous waste landfilled and incinerated in some of the countries found here at HRIHDW^3^. In Niger, Morocco, and Madagascar, 1,057,000, 58,810, and 33,812 tons, respectively, were landfilled, while 12,145, and 1698 tons, were incinerated in Madagascar and in Benin, respectively. Unregulated waste dumping sites have been identified as the source of Pb, Hg, Ni, Cu, Cr, Cd, and Zn in the main source of groundwater abstraction in southwestern Burkina Faso, where informal settlements and peri-urban agriculture place the population at risk^32^. Waste disposal and its incineration have been identified in Kinshasa, Democratic Republic Congo, among the main sources of Cd, Pb, and Ni in ambient air^33^. Both illegal and legal waste dumping sites, among other sources, have caused that HM such as Zn, As, and Pb are significantly present in sediment of Maqalika Reservoir, Lesotho. In particular, As and Pb were found in common carp fish at concentrations higher than the World Health Organization (WHO) permissible limits recommended for fish consumption, placing the residents at significant health risks from the intake of individual metals through fish consumption^34^.

Human and environmental damages produced by waste recycling are even more dramatic. In the case of e-waste, in most developing countries it is disposed of in domestic landfill sites and recycled in an informal way. This typically involves burning materials for recovering copper, and acid extraction to recover precious metals. Such practices are common in China, India, Pakistan, and Nigeria^35^, which are all identified here as countries at HRIHDW. As a consequence, in China^36^, levels of Pb in mother-infant pairs were found to be five times higher in regions known for the high concentration of e-waste disposal/processing than in control. It was associated to the higher rates of adverse birth outcomes observed in Guiyu—where 70% of global e-waste ends up^37^—related to control. In the same region children are reported to have significantly higher levels of Pb, Cr, and Ni, which have been linked to low mean intelligence coefficient (IQ), and decreased forced vital capacity^36^. Cd, Pb, Zn, Cu, Ni, As, and Cr were also found at higher levels in hairs of residents and dismantling workers in Longtang and Taizhou relative to control locations^36^. In India, it has been reported that the levels of dermal exposure of HM in workers of Indian e-waste recycling sites is 192.6 (Cr), 78.1 (Cu), 30.9 (Pb), and 37.3 (Zn) times higher than those for people not exposed to e-waste^38^.

In Nigeria, on average, 400,000 second-hand or scrap computers enter into the country annually, which represents about 60,000 metric tonnes per annum^39^. It is estimated that the country generates 1,100,000 tons of e-waste^3^. Several reports have quantified high levels of HM contamination of soils in e-waste dumpsites and of informal e-waste recycling in Nigeria^40–42^. Another waste-source of HM is the dismantling of used electric batteries, mainly from cars. Madagascar, which is one of the countries identified here at HRIHDW, is considered as a regular destination for traffic of batteries, where Pb is extracted by local scavengers and then send to China, Pakistan, or Dubai^43^. In Senegal, another country at HRIHDW, the death of 18 children^44^ has been linked to high levels of Pb in children living in surrounding areas used for recycling of used lead-acid batteries. In some cases, it is difficult to trace the HM to a particular country due to the transborder nature of the region contaminated. This is the case of the countries in the Gulf of Guinea—Benin, Cameroon, Ivory Cost, Ghana, Nigeria—where contamination by toxic waste dumping is known, which include high levels of heavy metals proceeding from e-waste^45,46^.

In Mexico, which is at HRIHDW, it has been reported 8 abandoned or illegal hazardous waste sites in Baja California and 15 ones in Coahuila, mainly containing HM. An estimated 6000 tons of Pb wastes, as well as other HM including Sb, As, Cd, and Cu resulting from the battery recycling operation have been reported in Tijuana, Baja California, Mexico^47^. In Djibouti, contamination of soils by As and Cr at concentrations 10 times higher than the US maximum contaminant levels where produced by a shipment of containers with up to 20 metric tons of chromate copper arsenate intended for treating electric poles, which were found leaking in the port of Djibouti^48^. In other countries found here at HRIHDW such as Papua New Guinea, Uzbekistan and Bangladesh there are reports of HM pollution affecting the environment and public health. However, it is difficult to trace these polluting HM to hazardous wastes due to the high impact of mining^49,50^ as well as industrial pollution^51,52^ in these countries.

### Volatile organic compounds

VOC are ubiquitous organic pollutants affecting atmospheric chemistry and human health^27^. VOC can be released from wastes containing solvents, paints, cleaners, degreasers, refrigerants, dyes, varnishes, and household wastes, from processing of e-wastes, plastics, and waste incineration^27,28^. We identify benzene (B), toluene (T), ethylbenzene (E), and o-, m-, and p-xylenes (X) as potential CF of Y1-Y47 waste^27,28,53,54^. Toluene is the only BTEX which has significant non-traffic sources, with important contributions from previously mentioned sources. Indeed, when the T/B ratio is over two it indicates the existence of waste sources beyond vehicular traffic^55^.

It is difficult to disentangle the possible sources for T/B ratios in the countries at HRIHDW to retain only the information concerning waste-generated VOC. For instance, in many developing countries T/B values can be affected by unofficial gasoline selling places, or by combustion processes for cooking in indoor kitchens. We have then searched for some potential sources of large T/B ratios that can be more likely be assigned to waste accumulation or processing. For instance, several VOC have been identified in an e-waste dismantling town in Guangdong province of China, including alkanes, BTEX, and organohalogen^53^. The T/B ratio found here was 3.15, which clearly correlates with emissions of VOC occurring during pyrolisis of e-waste^53^. T/B ratio of 9.36 is reported for Guangzhou^56^, which is the capital city of Guangdong. In the city of Dakar, Senegal, both at the urban district and at a semirural district, T/B ratios were 4.51 and 5.32^57^. Senegal is a country at HRIHDW for types I, II, and III. In Senegal, there have been continuous problems with the collection of household waste^58^, which have been responsible for public health problems (dermatosis, diarrhea, conjunctivitis, and malaria)^59^. Other HRIHDW countries with high values of T/B ratio reported at different locations are the following: Bangladesh (6.85), Benin (7.75), Burkina Faso (2.32), Ethiopia (2.3, 4.25), India (3.58, 3.67, 6.66, 8.97), Mexico (2.19, 5.70, 6.59) (see SI for references).

### Persistent organic pollutants

POP are chemicals with high resistance to degradation in the environment, high accumulation in human/animal tissues and transmission through food chains^29^. As POP indicators we consider here polychlorinated biphenyls (PCB)^60^ and polychlorinated dibenzo-p-dioxins and polychlorinated dibenzofurans (PCDD/Fs)^61^.

**Fig. 6:**
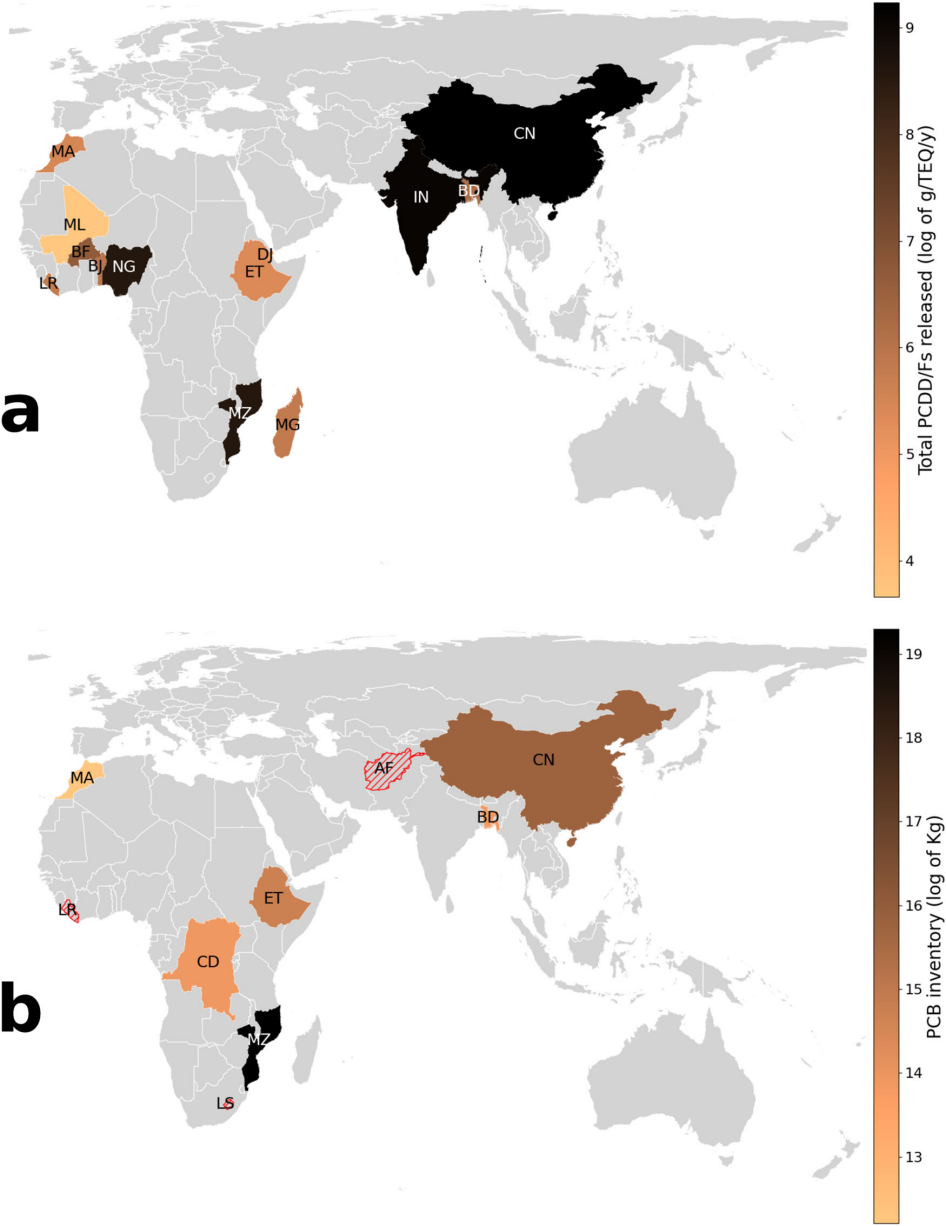
Geographical distribution of PCB and Dioxins. **a** Amounts of PCB stored in some of the countries at HRIHDW identified in this work. The amounts are given in logarithmic scale. **b** Total amounts of PCDD/Fs released to the environment by some of the countries at HRIHDW identified in this work. The amounts are given in logarithmic scale. The average amount of PCDD/Fs released in the 64 countries not in the list of countries at HRIHDW is 398.8 g/TEQ/y, which in log scale is 5.99. Map tiles by Bjorn Sandvik, under CC BY-SA 3.0 available at http://thematicmapping.org/downloads/world_borders.php.

PCB are intentionally produced due to their many industrial applications. They are related to neurodevelopment effects in infants, cancer, and immunotoxic effects in humans^60^. Vast amounts of PCB are stored in some of the countries at HRIHDW (Fig. 6a)^62^. For instance, in Mozambique 240,571 tonnes of oil suspected to have PCB are reported. Pollution by particulate and vapor samples containing PCB was detected in three sites in KwaZulu-Natal Province, South Africa^63^, which is close to the Mozambique border. PCBs are also found in four fish species from Lake Koka, Ethiopia^64^, where 2505 PCB-containing transformers with 1182 tons of PCB oil and 40 PCB-containing capacitors with 1.255 tons of PCB oil are reported. In China, high PCB concentrations have been reported in sediments from Pearl River and its estuary^65,66^. In Dalian Bay and Songhua River the pollution by PCB is directly related to PCB equipment storage locations^65^. In the Bengal coast of Bangladesh PCB contamination is linked to the past and on-going use of PCB-containing equipment^67^. Indeed, all 209 congeners of PCBs were found in 48 seafood samples collected from the coastal area of Bangladesh, with severe health risk for coastal residents^68^. In Bangladesh, it is known to exist 55.8 tons of PCB in use, 403 tons of contaminated oil contained in waste equipment, 519 tons of contaminated waste transformer oils and 22.5 tons of PCB contained in materials of old ships. Other three countries found here at HRIHDW—Lesotho, Liberia, and Morocco—have large amounts of equipments (transformers and condensers) containing PCB. In Morocco, for instance, 3500 tons of oil with more than 50 mg/kg of PCB are stored. There are 20 identured sites storing PCB wastes, from which 50% have been found to present floor pollution. Some African countries identified here at HRIHDW have also large storages of PCB waste. Nigeria has 341 transformers containing PCB. D. R. Congo has 188 PCB electrical transformers with 457 tons of PCB oils, 130,000 liters of pure PCB and 340,000 liters of PCB-containing oil and in Sierra Leone there are 103,372 tonnes of oil having PCB. The effects of PCB pollution in these countries have been documented. For instance, high levels of PCB has been found about 400 km off parts of the West African coast^69^. The total amount of PCB in the serum of recent immigrants who came from Sub-Saharan countries to the Canary Islands (Spain) identified levels of 78, 124, 141, 181, 181, 255 ng/g lipid for immigrants from Senegal, Guinea, Mali, Sierra Leone, D. R. Congo, and Nigeria, respectively^70^. Importantly, the authors of that report found that the immigrants’ PCB levels were strongly associated with the imports of second-hand e-waste by their country of origin, supporting our hypothesis connecting waste to CF and these to environmental/human damage.

On the other hand, PCDD/Fs are known to be extremely toxic in animals/humans^61^. Consequently, their release to the environment is presented as toxic equivalent (TEQ) (see Fig. 6b)^71^. In D. R. Congo alone PCDD/Fs amount to 300,412 g/TEQ/a (grams per toxic equivalent per year)^72^. It is followed by China (10,232), India (8658), Nigeria (5340), Lesotho (1708), and Sierra Leone (1242). The mean TEQ of PCDD/Fs in 75 countries, excluding those found here at HRIHDW, is 586.84, while that at HRIHDW is 2161.96^71^.

### Evolution of the W4 in the period 2001–2019

As we have seen the W4 has become slightly less densely connected from 2001 to 2019. The most dramatic change, however, has been registered by the drop of the average number of weighted triangles in the networks. This index has dropped an order of magnitude from 2002 to 2019. Here we are talking about directed triangles, that is, those in which a directed path A → B → C → A exists. Therefore, the number of directed triangles can decrease due to (i) the deletion of some of the edges forming the triangle, or (ii) due to the inversion of the direction of any of the three arrows of the triangle. The fact that we observe a positive trend in the clustering coefficient and a drop in edge density along the period, incline us to think more about the second possibility. In this scenario, there are three equivalent possibilities: (a) A ← B → C → A, (b) A → B → C ← A, and (c) A → B ← C → A. In the original triangle every country A, B, and C, has one input and one output, which make the system “balanced”, but in (a), (b), and (c), such balance is broken. In (a), the country A is becoming a net importer, while B is a net exporter. The same scenario is repeated for other nodes in (b) and (c). Although we focus here only on triangles the situation is repeated for other cycles as revealed by the fact that the weighted subgraph centrality of the countries also decay with time in this period.

The countries which displayed the most negative trend in the number of weighted triangles in the period 2001–2019 were Belgium, Germany, Netherlands, Spain, and France, closely followed by Ukraine, Canada, Ireland, and U.S. In order to investigate whether these countries have evolved to net importers or to net exporters, we use the difference △**S** = **S**_*in*_ − **S**_*out*_ of the in- **S**_*in*_ and out-strengths **S**_*out*_ of every node in the networks. The in-strength accounts for the weighted amounts of waste imported by a country, and the out-strength for those exported. We found that Germany, France, U.S., and Ukraine evolved from more balanced situations to become mainly net exporters in this period, with Pearson correlation coefficients *r* of △*S* vs. time of −0.78, −0.72, −0.66, and −0.44, respectively. Countries like Netherlands (*r* ≈ 0.88), Belgium (*r* ≈ 0.57), Spain (*r* ≈ 0.42), and Canada (*r* ≈ 0.34) evolved into net importers in the period.

In the general framework of the W4 evolution in the period 2011–2019, the countries displaying a more significant transformation to net exporters are Slovenia, U.K., New Zealand, and Germany, followed by France, and U.S. at different places among the top 15. On the other side, those transforming into net importers, the list is headed by Netherlands, Poland, Sweden, and R. Korea. Among the countries at HRIHDW the main transformation towards net exporter is observed in China (*r* ≈ −0.70) and those becoming major importers (in terms of the volume of waste) are Mexico (*r* ≈ 0.66), India (*r* ≈ 0.62), and Uzbekistan (*r* ≈ 0.47). In 15 of the countries at HRIHDW we found that *S*_*in*_ = 0 for every year reported. That is, these countries did not reported to have imported any waste during 2001–2019 but they export significant amounts of waste as to place them at HRIHDW. This situation in some of these countries could be a red alert of illegal waste imports which of course are not reported at the Basel documents (see Fig. 4 in ref. ^3^).

We also considered the betweenness centrality *BC* of the countries in the W4. The largest BC is observed in developed countries like U.K., France, Germany, Austria, Netherlands, and Belgium, while 116 countries, mainly developing ones, have BC equal to zero. We identified that 82.8% of the countries at HRIHDW have zero betweenness. That is, they are endpoints (net exporters or net importers) in the W4, while the developed countries have a more balanced situation in which imports and exports occur simultaneously.

We have introduced here a mathematical framework that allows to model the flows of waste through the international network of waste trade. This model allows to identify the time at which a given country reaches its carrying capacity and consequently it is considered to be congested or saturated of a given type of waste. Although we have merged several classes of waste into groups to facilitate the modeling and interpretation of results, the model can be adapted to individual waste categories according to BaC. Using this strategy we have identified countries which are at HRIHDW due to their relatively fast congestion of waste and their poor track record on environmental performance. The causes producing such potential waste saturation can be multiple and are not explored in this work. However, the current results trigger some red alert about the critical situation of some countries and the necessity of substantial investment in waste management at a global level.

The theoretical model presented here as well as the main results of this work can be applied to study the impact of different emerging scenarios, such as: (i) “Import bans” policies in major importers, like the one imposed in 2017 by China^73^. Consider a country *i* that exports HW to the set of countries *η*. If *j* ∈ *η* imposes a ban on HW, then the model can be used to redistributing the amount of wastes exported by *i* to a country *j* among the rest of countries in *η*. We can analyze how this redirectioning impacts on the congestion time of the countries in *η* and on the rest of the World;

(ii) Understanding the potential waste congestion problems arising from the COVID-19 pandemic and from emerging sources of e-waste^74,75^. Consider the set of countries that export/import a given type of waste, e.g., biomedical waste or e-waste. Increase those amounts in the model according to reported data or estimations, and calculate the congestion time for these countries as well as for the rest of the World;

(iii) Analyzing the impact of a global scenario of increasing amounts of all wastes. Use the model to proportionally increase the amounts of waste of every country/territory and run the simulation for determining the congestion time of every country, which can be compared with the results previous to the increase of waste amounts.

## Methods

### Data collection

We extract the data used to build W4 networks from BaC Online Reporting Database^76,77^. It contains summarized compendiums where individual national reports are altogether condensed into single Excel files per year, with the explicit and quantitative information of associated parties: destination, import, origin, and transit. We extract from these files the information about countries/territories of exports and imports, transaction amounts in metric tons, waste classification codes, characteristics, and type of waste streams. Code names of countries, and special territories like those that have no total political sovereignty, are considered by using the standard ISO 3166-1 alpha-2^78^. We do not include the countries of transit due to its scarcity in the reports, and because of the lack of information about the temporary order of the landings. We also excluded the existing self-export (a country that exports to itself). We manually curated the database for errors in the country/territories names, e.g., due to typos or possible transcription errors, as well as for the use of nonofficial country codes such as EIRE instead of IE for Ireland. The BaC reports may also combine formal ISO alpha-2 codes with others codes that have become obsolete and sometimes with codes of another standards like transitory codes or international postal union codes. Reports may pointing out to a state party that currently is dissolved or split into two new ones, e.g., Serbia and Montenegro. In the case of waste categories, we also exclude those for which their codes do not coincide with the ones defined by the BaC, such as 11b, AN8, Y48.

### Waste types

We consider 108 categories of wastes according to BaC classification, which are then grouped into seven types of waste designated by Type I–VII. The classification of wastes used in this work is based on the Annexes I, II, and VII of the BaC^76^. No wastes in category B of the BaC are included in this work as they are not reported by countries in the database of the Convention^77^.

Type I considers, for instance, Y1: Clinical wastes from medical care in hospitals, medical centers of clinics, Y2: Wastes from the production and preparation of pharmaceutical products, up to Y18: Residues arising from industrial waste disposal operations (see pp. 46 of ref. ^76^). The Type II of wastes used in this work associates the second subdivision of the Annex I, Y-codes Y19-Y45. In general, wastes containing 27 chemical constituents, i.e., Y19: Metal carbonyls, Y20: Beryllium compounds, up to Y45: Organohalogen compounds. The type III of wastes discussed here accounts for the Annex II of the BaC classification. Y46: Wastes collected from households, and Y47: Residues arising from the incineration of household wastes. A complete list is provided in the SI.

The remaining four types of wastes recover the four subclassifications of the Annex VIII^76^. Specifically, Type IV links with the Metal and Metal-Bearing Wastes. It accounts for A-list items grouped from A1010 to A1090 and A1100 to A1190, e.g., A1010: Metal wastes and waste consisting of alloys of Antimony, Arsenic, Cadmium, Selenium, among others; up to A1190: Waste metal cables coated or insulated with plastics containing or contaminated with coal tar, PCB11, lead, cadmium, other organohalogen compounds (see pp. 66 of ref. ^76^). Type V relates Inorganic constituents containing metal and organic material (cathode-ray glasses, liquid inorganic fluorines, catalysts, gypsum, dust-fibers of asbestos, coal-fired power plant fly-ash). Its A-items ranges from A2010 to A2060. Type VI associates Organic constituents containing metal and inorganic material. (Petroleum coke and bitumen, mineral oils, leaded anti-knock sludge, thermal fluids, resin, latex, plasticizers, glues, adhesives, nitrocellulose, phenols, ethers, leather wastes, (un)halogenated residues, aliphatic halogenated hydrocarbons, vinyl chlorides), accounting for A-items: A3010–A3090, A3100–A3190, and A3200. Finally, Type VII are Wastes which may contain either inorganic or organic constituents (Some pharmaceutical products, clinical-medical-nursing-dental-veterinary wastes from patients and researches, biocides-phytopharmaceutical, pesticides, herbicides outdated, wood chemicals, (in)organic cyanides, oils-hydrocarbons-water mixtures, inks, dyes, pigments, paints, lacquers, varnish, of explosive nature, industrial pollution control devices, for cleaning of industrial off-gases, peroxides, outdated chemicals, from research or teaching activities, spent activated carbon, to name a few). It accounts for the groups A4010–A4090 and A4100–A4160.

### W4 construction

We construct a weighted-directed network for each of the types of waste analyzed. In every network, the nodes correspond to the countries/territories reporting the given type of waste in the period 2001–2019. It is frequent in the BaC database that a country *i* reports the export (import) of an amount *q*_*ij*_ to (from) *j*, which includes several BaC waste categories. If all the BaC categories belong to the same waste type, then we simply use that amount as the weight of the link (*i*, *j*). However, it happens sometimes these BaC categories belong to several waste types. Let us consider two BaC categories *C*_1_ and *C*_2_, e.g., Y1 and Y19. Then, *C*_1_ belongs to one waste type, e.g., type I, and *C*_2_ to another, e.g., type II. In this case, we have to split the quantity *q*_*ij*_ in the weights of the links between *i* and *j* for the two types of wastes. We then proceed as follows. We obtain the weight of the link (*i*, *j*) for the waste of type *k* as

8 $${w}_{ij}^{k}=\frac{{q}_{ij}\cdot {\phi }_{k}}{{{\Phi }}},$$

where *ϕ*_*k*_ is the average of the amounts of waste of type *k* traded between every pair of countries during the corresponding year, and Φ = ∑_*k*_ *ϕ*_*k*_ where the summation is carried out for all types of waste involved in the quantity *q*_*ij*_.

In any case, we can obtain two different weights for a pair of countries based on the data reported at BaC from “Export” and “Import” reports. Then we can have the following two different cases: (a) that the amount $E\left(i,j\right)$ reported by country *i* as exported to country *j* coincides with the amount $I\left(j,i\right)$ reported by *j* as imported from *i*; (b) that $E\left(i,j\right)\ne I\left(j,i\right)$. In the case (a), we simply add a directed arc from *i* to *j* with the weight $E\left(i,j\right)=I\left(j,i\right).$ In the case (b), we assume that *i* exports $\max \left[E\left(i,j\right),I\left(j,i\right)\right]$ to *j*. We designate by $\tilde{A}=\tilde{A}\left(G\right)$ the adjacency matrix of the network *G*. Notice that $\tilde{A}$ is not necessarily symmetric because ${\tilde{A}}_{ij}=\max \left[E\left(i,j\right),I\left(j,i\right)\right]$ is not necessarily the same as ${\tilde{A}}_{ji}=\max \left[E\left(j,i\right),I\left(i,j\right)\right]$. Here we normalize the adjacency matrices by $A=\tilde{A}/{\sum }_{i,j}{\tilde{A}}_{ij}$.

### Network parameters of the W4 networks

Because the W4 networks are weighted and directed we consider here the distributions of their in- and out-strengths, **S**_*in*_ and **S**_*out*_, respectively. The in-strengths of the node *i* is the sum of the weights of all links pointing to *i*. The out-strength of that node is the sum of the weights of all links leaving that node. For each kind of strength, we tested 17 types of distributions^79,80^: beta, Birnbaum-Saunders, exponential, extreme value, gamma, generalized extreme value, generalized Pareto, inverse Gaussian, logistic, log-logistic, lognormal, Nakagami, normal, Rayleigh, Rician, *t*-location-scale, and Weibull. To test the goodness of fit we used^81^: negative of the log-likelihood, Bayesian information criterion, Akaike information criterion (AIC), and AIC with a correction for finite sample sizes. The results are given in the Supplementary Information.

For the global characterization of the W4 networks we used the following structural parameters (see ref. ^82^):

(i) Edge density, $$\delta \left(G\right)$$
  9 $$\delta \left(G\right):=\frac{m}{n\left(n-1\right)},$$
  where *n* is the number of nodes and *m* is the number of directed edges;
(ii) Reciprocity, $$\rho \left(G\right)$$
  10 $$\rho \left(G\right):=\frac{r-\bar{a}}{1-\bar{a}},$$
  where the network is binarized before the calculation, $\bar{a}$ measures the ratio of observed to possible directed links and
  11 $$r=\frac{{L}^{\leftrightarrow }}{m},$$
  with *L*^↔^ being the number of reciprocal edges and *m* the total number of directed edges (see ref. ^83^ for details);
(iii) Average number of weighted-directed triangles, $\bar{t}$
  12 $$\bar{t}:=\frac{tr\left({A}^{3}\right)}{3n},$$
  where $tr\left(.\right)$ is the trace of the matrix *A*, and *A* is the adjacency matrix of the network;
(iv) Average clustering coefficient, $\bar{C}$
  13 $$\bar{C}:=\frac{1}{n}\mathop{\sum }\limits_{i=1}^{n}\frac{2{t}_{i}}{{k}_{i}^{tot}\left({k}_{i}^{tot}-1\right)-2{k}_{i}^{\leftrightarrow }},$$
  where *t*_*i*_ is the number of directed triangles through node *i*, ${k}_{i}^{tot}$ is the sum of in- and out-degree of the node *i*, and ${k}_{i}^{\leftrightarrow }$ is the reciprocal degree of *i* (see ref. ^84^ for details);
(v) Average path length, $\bar{l}$
  14 $$\bar{l}:=\frac{1}{n\left(n-1\right)}\mathop{\sum}\limits_{i,j}{l}_{ij},$$
  where *l*_*ij*_ is the length of the shortest path distance between the nodes *i* and *j*, where in case that there is not a path between two nodes the value of zero is assigned to its length;
(vi) Average subgraph centrality, $\bar{{S}_{C}}$
  15 $$\bar{{S}_{C}}:=\frac{1}{n}tr\left({e}^{A}\right),$$
  where $\exp \left(A\right)$ is the matrix exponential of the adjacency matrix.

For the analysis of the individual countries of the network, apart from the in- and out-strengths, we also analyze the following local measures of W4^79^. We refer the indices to a node labeled as *i*:

(i) Number of weighted-directed triangles
  16 $${t}_{i}:={\left({A}^{3}\right)}_{ii};$$
(ii) Subgraph centrality
  17 $$S{C}_{i}:={\left({e}^{A}\right)}_{ii};$$
(iii) Betweenness centrality
  18 $$B{C}_{i}:=\mathop{\sum}\limits_{p\ne i\ne q}\frac{{\varrho }_{piq}}{{\varrho }_{pq}},$$
  where *ϱ*_*piq*_ is the number of weighted-directed shortest paths from *p* to *q* that goes through the node *i* and *ϱ*_*pq*_ is the total number of weighted-directed shortest paths from *p* to *q*.

### Congestion dynamics

We consider the same rate for both, congestion at arrival and congestion at departure, processes, i.e., *β*_*A*_ = *β*_*D*_. In the simulations we use *α* = 0.75, *β* = 0.01, and *c* = 0.005. As a way of quantifying how easily a country gets congested by a given waste we use the time at which 50% of the total congestion is reached, which is more accurate to determine that the time at which 100% of saturation is reached. Let us designate by ${\hat{t}}_{i}$ this time. Then, ${\hat{t}}_{i}$ is the time *t* at which ${s}_{\ell }\left(t\right)=0.5$.

We will illustrate the intuition behind the use of separated equations for congestion at arrival and at departure with an example. Let us consider a trade network of three countries where *A* exports 100 tonnes of waste to *B* and 120 tonnes to C; *B* exports 200 tonnes to *C*, and *C* exports 50 tonnes to *A*. Let the times ${\hat{t}}_{C} \; < \; {\hat{t}}_{A}\; < \;{\hat{t}}_{B}$ for the congestion at arrival model (see SI Fig. [8] for graphic illustration).

This indicates that *C* is at the highest risk of congestion due to its large imports of waste. However, if we consider the process at departure, ${\hat{t}}_{A}\; < \;{\hat{t}}_{B}\; < \;{\hat{t}}_{C}$, which indicates the highest risk at node *A* due to the existence of large amounts of this waste at the node.

### Potential environmental impact of waste congestion

We first define here the risk of waste congestion for a given country as

19 $${R}_{i}:=1-{\hat{t}}_{i}/\mathop{\max }\limits_{j}{\hat{t}}_{j},$$

where *i* represents a given country, ${\hat{t}}_{i}$ is the congestion time for the country *i* either by importing or by exporting wastes of a given type. That is, if ${t}_{1/2}\left(i\leftarrow \right)$ and ${t}_{1/2}\left(i\to \right)$ are the times at which country *i* reaches 50% of congestion by importing and exporting a given type of waste, respectively, then ${\hat{t}}_{i}=\min [{t}_{1/2}\left(i\leftarrow \right),{t}_{1/2}\left(i\to \right)]$. The index *R*_*i*_ is normalized between zero (no risk) and one (maximum risk) of congestion of wastes of a given type.

Due to the socio-economic differences between the countries in the world, the use of *R*_*i*_ along could be of little practical value. For instance, for wastes of type I the Netherlands and Burkina Faso have about the same value of *R*_*i*_, which is near 0.99. For the same type of wastes Ireland and Ivory Cost also have *R*_*i*_ ≈ 0.89. The situation is similar for waste of type II, where the first pair of countries have *R*_*i*_ = 1 and the second pair have *R*_*i*_ ≈ 0.94. However, while Netherlands and Ireland are among the richest countries in the world with GDP ranging 578–868 billions USD (Netherlands) and 164–236 billions USD (Ireland), the other two countries are among the poorest with GDPs of 4.7–9.4 billions USD (Burkina Faso) and 15–24 billions USD (Ivory Cost) for the period of time considered here. This obviously gives these countries very different capacities for managing a waste congestion, a situation which is well reflected in the environmental track record of each of these countries. The Environmental Performance Index (EPI), published by the Universities of Yale and Columbia^25^, quantifies the performance of every country using sixteen indicators reflecting United Nations’ Millennium Development Goals. They are accounted for by six well-established policy categories (see Policymakers’ Summary at ref. ^25^): Environmental Health, Air Quality, Water Resources, Productive Natural Resources, Biodiversity and Habitat, and Sustainable Energy, such that it covers the following two global goals: (1) reducing environmental stresses on human health, and (2) promoting ecosystem vitality and sound natural resource management. Then, while Netherlands and Ireland are among the top environmental performers in the 2001–2019 period with average EPIs larger than 70 out of 100, Burkina Faso and Ivory Cost are the bottom of the list with average EPIs of 45.2 and 55.9, respectively. We can account the risk of environmental underperformance by an index bounded between zero and one as: ${U}_{i}=1-{{{{{\rm{EPI}}}}}}\left(i\right)/100$. PEIWC are defined by plotting the waste congestion risk *R*_*i*_ for a given type of waste versus *U*_*i*_. For the demarcation of the tolerance zone we use here the following. We obtain the linear regression model that best fit *U*_*i*_ as a linear function of *R*_*i*_. Then, the tolerance zone is defined by the upper and lower 50% prediction bounds for response values associated with this linear regression trend between the two risk indices. The value of 50% is used here as a very conservative definition of the tolerance zone. Widening this zone too much will make that almost no country is at HRIHDW, which does not reflect the reality. On the contrary, narrowing it too much will simply split countries into two classes, which will make it difficult to identify those at the highest risk of environmental underperformance due to waste congestion. Although we have identified 58 countries over this tolerance zone, i.e., the countries at HRIHDW, we performed our studies on a subset of them formed by 28 countries. These countries, referred in the text as “top 28”, were selected by picking in every PEIWC those countries in the top 25% of deviation over the tolerance zone. We then merged the sets of countries found at every PEIWC conforming this list of top 28 at HRIHDW.

## Supplementary information

Supplementary Information

## Data Availability

Extracted set of Export and Import networks generated in this study is available in https://github.com/JohannHM/Fractional-congestion-Dynamics. J.H. Martínez, S. Romero, J.J. Ramasco, E. Estrada, The world-wide waste web. JohannHM/Fractional-congestion-Dynamics: The World-wide waste web. Data and code (v1.0.0). Zenodo. 10.5281/zenodo.5786874 (2021). All raw data of the manuscript and its Supplementary Information was obtained directly from the Basel Convention web page: http://www.basel.int/Countries/NationalReporting/ElectronicReportingSystem/tabid/3356/Default.aspx.

## Code availability

Custom MATLAB code is available on GitHub (https://github.com/JohannHM/Fractional-congestion-Dynamics). J.H. Martínez, S. Romero, J.J. Ramasco, E. Estrada, The world-wide waste web. JohannHM/Fractional-congestion-Dynamics: The World-wide waste web. Data and code (v1.0.0). Zenodo. 10.5281/zenodo.5786874 (2021).
