# Supplementary material for: The world-wide waste web
Source: Nat Commun. 2022 Mar 29;13:1615. doi: 10.1038/s41467-022-28810-x (PMC8964736; doi:10.1038/s41467-022-28810-x)
Supplement: Supplementary file 1 — Supplementary Information [file 41467_2022_28810_MOESM1_ESM.pdf]

Supporting Information for "The world-wide waste web"

Johann H. Martínez, Sergi Romero, José J. Ramasco and Ernesto Estrada

This material contains relevant innformation about supplementary dicussion and supplementary mehtods related to the connectivity distribution of the w4 and different types of network centralities; supplementary data, tables and figures with specifications of the chemical fingerprints, as well as for the waste classificacion.

Supplementary information guide

- Degree distributions of W4 networks.
- Chemical fingerprints common in hazardous waste.
- Centrality of countries in the W4 networks.
- Susceptible-waste congested model.
- Chemical fingerprints.
- Countries/territories without EPI.
- Waste categories in types IV-VII. PEIWS analysis of wastes types IV-VII.

Degree distributions of W4 networks

For each of the type I-III we calculated the in- and out-strengths (weighted degrees) of its nodes and tested 17 probability distribution functions: beta, Birnbaum-Saunders, exponential, extreme value, gamma, generalized extreme value, generalized Pareto, inverse Gaussian, logistic, log-logistic, lognormal, Nakagami, normal, Rayleigh, Rician, t-location-scale, and Weibull. The goodness of fit is tested by calculating the following parameters: negative of the log likelihood (NlogL), Bayesian information criterion (BIC), Akaike information criterion (AIC), and AIC with a correction for finite sample sizes (AICc). The results are as follows.

Waste type I

No.	distribution	NlogL	BIC	AIC	AICc
1	generalized pareto	-3249.47	-6483.38	-6492.94	-6492.80
2	generalized extreme value	-2196.84	-4378.12	-4387.68	-4387.54
3	t-location scale	-1548.53	-3081.49	-3091.05	-3090.91
4	exponential	-749.54	-1493.90	-1497.08	-1497.06
5	logistic	-535.26	-1060.14	-1066.51	-1066.45
6	normal	-371.55	-732.72	-739.10	-739.03
7	extreme value	-206.90	-403.42	-409.80	-409.73

Table SI. 1: Values of the statistical parameters quantifying the goodness of fit of the distributions fitting the in-strength of the nodes of the W4 network of wastes type I. Only the top seven distributions are shown.

No.	distribution	NlogL	BIC	AIC	AICc
1	generalized extreme value	-1298.60	-2581.63	-2591.20	-2591.06
2	generalized pareto	-1291.81	-2568.05	-2577.62	-2577.48
3	t-location scale	-1166.20	-2316.85	-2326.41	-2326.27
4	beta	-1012.85	-2015.33	-2021.70	-2021.63
5	exponential	-749.54	-1493.90	-1497.08	-1497.06
6	logistic	-542.04	-1073.71	-1080.09	-1080.02
7	normal	-433.29	-856.20	-862.57	-862.50

Table SI. 2: Values of the statistical parameters quantifying the goodness of fit of the distributions fitting the out-strength of the nodes of the W4 network of wastes type I. Only the top seven distributions are shown.

Waste type II

No.	distribution	NlogL	BIC	AIC	AICc
1	generalized pareto	-3088.32	-6161.10	-6170.63	-6170.49
2	generalized extreme value	-1764.62	-3513.72	-3523.25	-3523.11
3	t-location scale	-1386.06	-2756.60	-2766.13	-2765.99
4	exponential	-739.19	-1473.18	-1476.36	-1476.33
5	logistic	-535.85	-1061.34	-1067.69	-1067.63
6	normal	-422.29	-834.22	-840.57	-840.50
7	extreme value	-287.85	-565.35	-571.71	-571.64

Table SI. 3: Values of the statistical parameters quantifying the goodness of fit of the distributions fitting the in-strength of the nodes of the W4 network of wastes type II. Only the top seven distributions are shown.

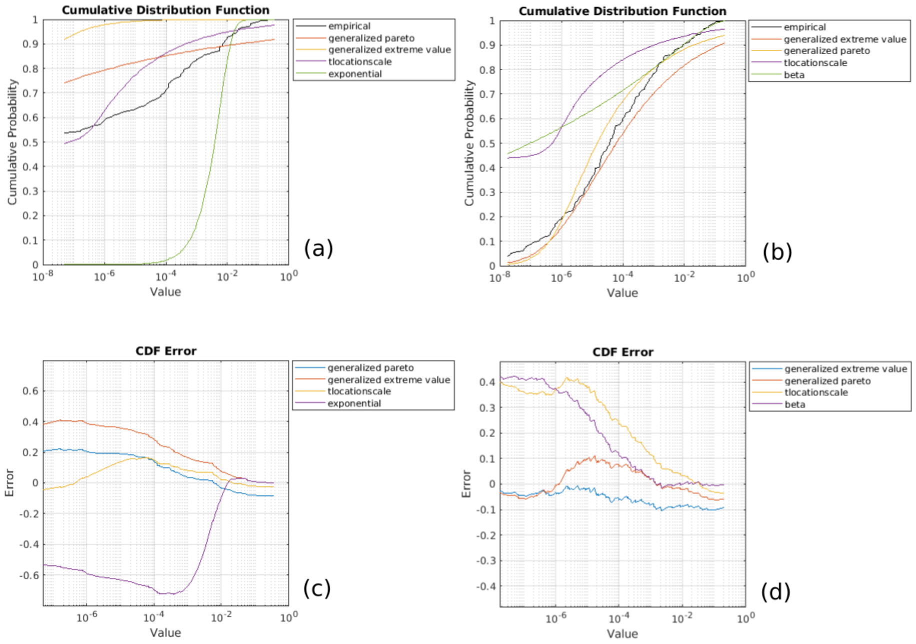

Figure SI. 1: Cumulative in- (a) and out-degree (b) distributions and the corresponding errors (c)-(d) for the W4 of type I wastes. The empirical distribution refers to the data of the W4 network and the others correspond to the best fits using different kinds of distributions, e.g., lognormal, loglogistic, generalized extreme value, etc. The errors are obtained as the differences of the empirical values of the degrees and those estimated by the different kinds of distributions.

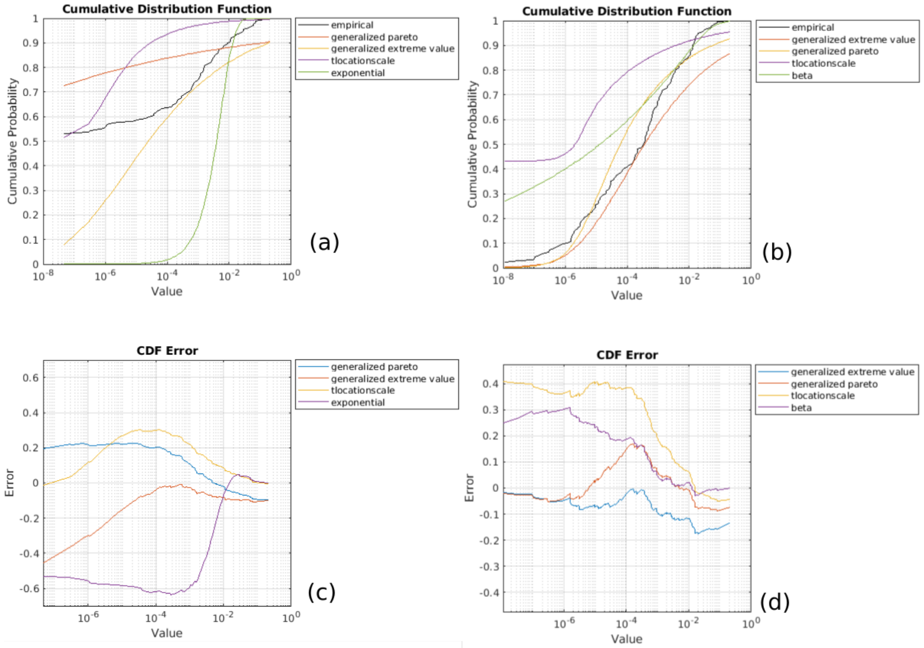

Figure SI. 2: Cumulative in- (a) and out-degree (b) distributions and the corresponding errors (c)-(d) for the W4 of type II wastes. The empirical distribution refers to the data of the W4 network and the others correspond to the best fits using different kinds of distributions, e.g., lognormal, loglogistic, generalized extreme value, etc. The errors are obtained as the differences of the empirical values of the degrees and those estimated by the different kinds of distributions.

No.	distribution	NlogL	BIC	AIC	AICc
1	generalized extreme value	-1051.89	-2088.25	-2097.78	-2097.64
2	generalized pareto	-1048.39	-2081.26	-2090.78	-2090.64
3	t-location scale	-925.87	-1836.21	-1845.74	-1845.60
4	beta	-896.47	-1782.60	-1788.95	-1788.88
5	exponential	-739.18	-1473.18	-1476.36	-1476.33
6	logistic	-555.88	-1101.41	-1107.76	-1107.69
7	normal	-444.52	-878.68	-885.04	-884.97

Table SI. 4: Values of the statistical parameters quantifying the goodness of fit of the distributions fitting the out-strength of the nodes of the W4 network of wastes type II. Only the top seven distributions are shown.

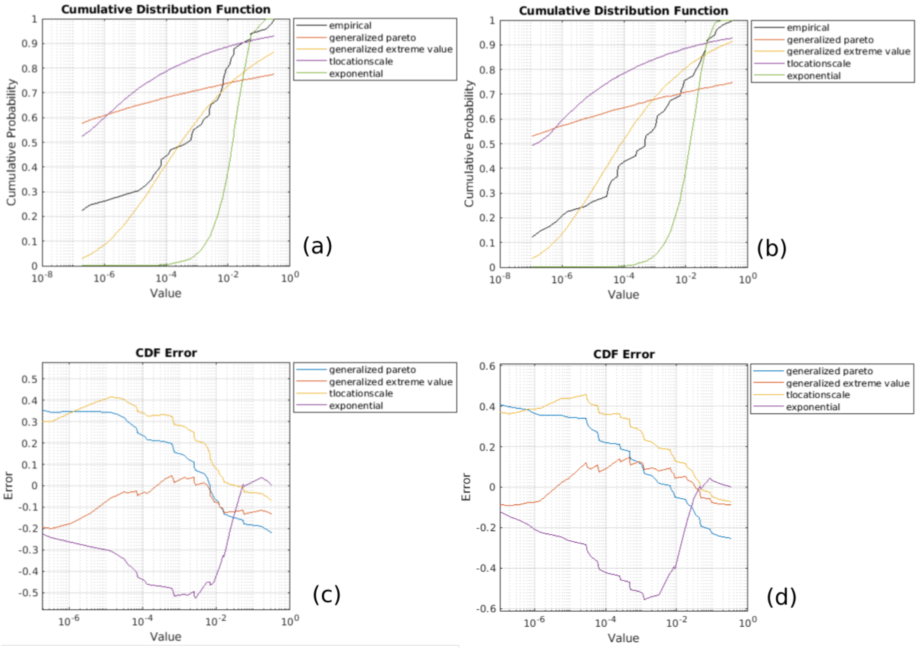

Figure SI. 3: Cumulative in- (a) and out-degree (b) distributions and the corresponding errors (c)-(d) for the W4 of type III wastes. The empirical distribution refers to the data of the W4 network and the others correspond to the best fits using different kinds of distributions, e.g., lognormal, loglogistic, generalized extreme value, etc. The errors are obtained as the differences of the empirical values of the degrees and those estimated by the different kinds of distributions.

No.	distribution	NlogL	BIC	AIC	AICc
1	generalized pareto	-405.46	-799.25	-804.93	-804.40
2	generalized extreme value	-279.99	-548.31	-553.99	-553.45
3	t-location scale	-241.62	-471.56	-477.23	-476.70
4	exponential	-141.70	-279.51	-281.40	-281.31
5	logistic	-88.38	-168.98	-172.77	-172.51
6	normal	-66.97	-126.15	-129.94	-129.68
7	beta	-54.64	-101.50	-105.29	-105.02

Table SI. 5: Values of the statistical parameters quantifying the goodness of fit of the distributions fitting the in-strength of the nodes of the W4 network of wastes type III. Only the top seven distributions are shown.

No.	distribution	NlogL	BIC	AIC	AICc
1	generalized pareto	-292.38	-573.10	-578.77	-578.24
2	generalized extreme value	-264.19	-516.70	-522.38	-521.85
3	t-location scale	-222.87	-434.06	-439.73	-439.20
4	exponential	-141.70	-279.51	-281.40	-281.31
5	beta	-116.92	-226.06	-229.84	-229.58
6	logistic	-92.51	-177.24	-181.03	-180.77
7	normal	-72.19	-136.60	-140.38	-140.12

Table SI. 6: Values of the statistical parameters quantifying the goodness of fit of the distributions fitting the out-strength of the nodes of the W4 network of wastes type III. Only the top seven distributions are shown.

Waste type III

Chemical fingerprints common in hazardous waste

It has been argued that all chemical substances which are outside their usual environments or at concentrations above normal represent a contaminant and that they become pollutants when accumulations are sufficient to affect the environment or living organisms.[1] The sources of these chemicals may be very diverse, but some of the top contaminants emerge from waste. These are the cases of heavy metals, such as arsenic, lead, mercury, hexavalent chromium, and cadmium; volatile organic compounds like vinylchloride, benzene, hexachlorobutadiene or persistent organic pollutants like polycyclic aromatic compounds, polychlorinated biphenyls and dioxins.[1] The distribution of contaminants across the world is very unequal with a bigger impact on low and middle-income countries.[2] In these countries there are serious problems with the disposal of waste, which is poorly managed, regulated or controlled,[2] and obsolete techniques are applied for their processing, which together with the lack of governmental infrastructure makes the situation critical.[2] The substantial inform "Chemical

Pollution in Low and Middle-Income Countries"[2] has identified many of the problems emerging in these countries due to the chemical pollution in spite of the many internationally existing legislation, such as the Stockholm, Basel, and Rotterdam Conventions, and the Strategic Approach to International Chemicals Management (SAICM).

Generally, the term "hazardous" waste (HW) is used to define waste with potential threats to public health[3] and the environment. It includes waste electrical and electronic equipment, commonly designated as e-waste, which has become the fastest-growing component of the solid-waste streams in the world.[4] E-waste disposal and its informal management generates highly toxic heavy metals, brominated flame retardants, non-dioxin-like polychlorinated biphenyls (PCB), polycyclic aromatic hydrocarbons (PAH), polychlorinated dibenzo-p-dioxins (PCDD), polychlorinated dibenzofurans (PBDF) and dioxin-like polychlorinated biphenyls (DL-PCB). These compounds are endocrine disrupters, and most are neuro- and immune-toxic as well. Another important source of chemical contamination is the clinical and medical waste.[5–7] Some attention has been given to the cases of infection transmission due to the inappropriate disposal and handling of this type of waste,[5–7] but less is known about the chemical traces left by medical waste on the environment and human populations. Another growing source of chemical contaminants is municipal solid waste, which tripled from 1965 to 2015.[8]

Waste problems in developing countries is aggravated by the transboundary trade of HW, which increases their burden of certain kinds of waste in those countries.[9–13] The problem should be analyzed from a wide perspective. Namely, we do not claim here that waste trade is the source of all the environmental and human health problems that waste produces. We claim that in those countries with a large burden due to bad practices and poor resources for waste management, importing any amount of waste will only aggravate their situation. This is illustrated by the fact that in Africa there are significant levels of soil pollution due to agricultural activities, mining, roadside emissions, automechanic workshops, their own refuse dumps and e-waste.[14] Then, when poor African countries like Nigeria, become a dumping ground for HW imported from abroad,[15] the situation become of a very high risk. In Africa there are 67,740 health-care facilities which generate 56,100-487,100 tonnes of medical waste per year. These global amounts are not exaggeratedly large, but the risk is very high considering that medical waste is rarely sorted which makes the amounts of HW much higher than in other parts of the world.[16,17] Then, when these countries receive large amounts of e-waste from abroad they do not have resources for dealing with its recycling. The consequences are elevated levels of e-waste pollutants in water, air, soil, dust, fish, vegetable, and human blood, urine, breast milk, producing headache, cough and chest pain, stomach discomfort, miscarriage, abnormal thyroid and reproductive function, reduction of gonadal hormone, and cancer in those involved with the processing of e-waste.[18]

In Table SI. 7 we report the chemical fingerprints left by different types of waste according to an intensive bibliographic search carried out in this work. In Tables SI. 8, SI. 9 and SI. 10 we then report every individual waste category

reported by the Basel Convention, which are on the types I-III considered in this work. We report the chemical fingerprints left by these waste based on the specific report on their contents at the Basel Convention web page.

fingerprint	wastes	ref.
HM	pesticides, paints and pigments, enamel, varnishes, dyes, catalysts, batteries, accumulators, printing products, e-waste, metal products, asbestos, anticorrosive, technical oils, sewage sludge, waste incineration products, waste from plastic production, PVC plastics, colored glass, glues, ash of coal, metalurgical slag, galvanic waste, waste of nonferrous metallurgy, waste of leather industry, agriculture waste, waste of medicines, medical/clinical waste	[19–26]
VOC	solvents, wastes from petroleum refining, synthetic resin, textile dyeing and printing, leather manufacturing, the pharmaceutical industry, pesticide manufacturing, coating, printing ink, adhesive manufacturing, spraying, printing, e-waste, plastic solid waste recycling, municipal solid waste, agricultural waste burning	[21,22,27–33]
POP	municipal solid waste, medical waste, sewage sludge, and hazardous waste incinerations; informal recycling of e-waste; drilling wastes; PCB-containing additives in rubber, resins, carbonless copy paper, inks, hydraulic fluids, heat-transfer fluids, plasticizers, and lubricants; PCB-containing transformers, capacitors; PCB-containing wastes from coating of papers, sealants of cars, coloring of China glassware, color television parts, the effect extension agents of agricultural chemicals, oil additive agents	[21,22,25,34–38]

Table SI. 7: Chemical fingerprints left by waste in the environment and/or human health. The fingerprints are classified as heavy metals (HM), volatile organic compounds (VOC) and persistent organic pollutants (POP).

Waste categories in types I-III

category	description	fingerprints
Y1	Clinical wastes from medical care in hospitals, medical centers and clinics	HM, VOC, POP
Y2	Wastes from the production and preparation of pharmaceutical products	HM, VOC, POP
Y3	Waste pharmaceuticals, drugs and medicines	HM, VOC, POP
Y4	Wastes from the production, formulation and use of biocides and phytopharmaceuticals	HM, VOC, POP
Y5	Wastes from the manufacture, formulation and use of wood preserving chemicals	HM, VOC, POP
Y6	Wastes from the production, formulation and use of organic solvents	VOC
Y7	Wastes from heat treatment and tempering operations containing cyanides	VOC
Y8	Waste mineral oils unfit for their originally intended use	VOC, POP
Y9	Waste oils/water, hydrocarbons/water mixtures, emulsions	VOC, POP
Y10	Waste substances and articles containing or contaminated with polychlorinated biphenyls (PCBs) and/or polychlorinated terphenyls (PCTs) and/or polybrominated biphenyls (PBBs)	POP
Y11	Waste tarry residues arising from refining, distillation and any pyrolytic treatment	HM, VOC, POP
Y12	Wastes from production, formulation and use of inks, dyes, pigments, paints, lacquers, varnish	VOC, POP
Y13	Wastes from production, formulation and use of resins, latex, plasticizers, glues/adhesives	VOC, POP
Y14	Waste chemical substances arising from research and development or teaching activities which are not identified and/or are new and whose effects on man and/or the environment are not known	HM, VOC, POP
Y15	Wastes of an explosive nature not subject to other legislation	VOC
Y16	Wastes from production, formulation and use of photographic chemicals and processing materials	HM, VOC, POP
Y17	Wastes resulting from surface treatment of metals and plastics	VOC, POP
Y18	Residues arising from industrial waste disposal operations	HM, VOC, POP

Table SI. 8: Waste categories included in the Basel Convention which are grouped in the type I of wastes and the chemical fingerprints (CF) left by them in the environment and/or human health: heavy metals (HM), volatile organic compounds (VOC) and persistent organic pollutants (POP).

category	description	fingerprints
Y19	Metal carbonyls	HM, VOC
Y20	Beryllium; beryllium compounds	HM
Y21	Hexavalent chromium compounds	HM
Y22	Copper compounds	HM, VOC, POP
Y23	Zinc compounds	HM
Y24	Arsenic; arsenic compounds	HM
Y25	Selenium; selenium compounds	HM
Y26	Cadmium; cadmium compounds	HM
Y27	Antimony; antimony compounds	HM
Y28	Tellurium; tellurium compounds	HM
Y29	Mercury; mercury compounds	HM
Y30	Thallium; thallium compounds	HM
Y31	Lead; lead compounds	HM
Y32	Inorganic fluorine compounds excluding calcium fluoride	HM
Y33	Inorganic cyanides	VOC
Y34	Acidic solutions or acids in solid form	VOC, POP
Y35	Basic solutions or bases in solid form	VOC, POP
Y36	Asbestos (dust and fibres)	
Y37	Organic phosphorus compounds	VOC, POP
Y38	Organic cyanides	VOC
Y39	Phenols; phenol compounds including chlorophenols	VOC
Y40	Ethers	VOC
Y41	Halogenated organic solvents	VOC, POP
Y42	Organic solvents excluding halogenated solvents	VOC
Y43	Any congenor of polychlorinated dibenzo-furan	POP
Y44	Any congenor of polychlorinated dibenzo-p-dioxin	POP
Y45	Organohalogen compounds other than substances referred to in this Annex (e.g. Y39, Y41, Y42, Y43, Y44)	HM, VOC, POP

Table SI. 9: Waste categories included in the Basel Convention which are grouped in the type II of wastes and the chemical fingerprints (CF) left by them in the environment and/or human health: heavy metals (HM), volatile organic compounds (VOC) and persistent organic pollutants (POP).

category	description	fingerprints
Y46	Wastes collected from households	HM, VOC, POP
Y47	Residues arising from the incineration of household wastes	HM, VOC, POP

Table SI. 10: Waste categories included in the Basel Convention which are grouped in the type III of wastes and the chemical fingerprints (CF) left by them in the environment and/or human health: heavy metals (HM), volatile organic compounds (VOC) and persistent organic pollutants (POP).

Centrality of countries in the W4 networks

Here we introduce other networks metrics for waste types I, II, and III. Fig. SI. 4 depicts the in-/out-strengths. Fig. SI. 5 shows the betweenness centrality, and Fig. SI. 6 introduces the in-closeness for types I, II, III; and the out-closeness.

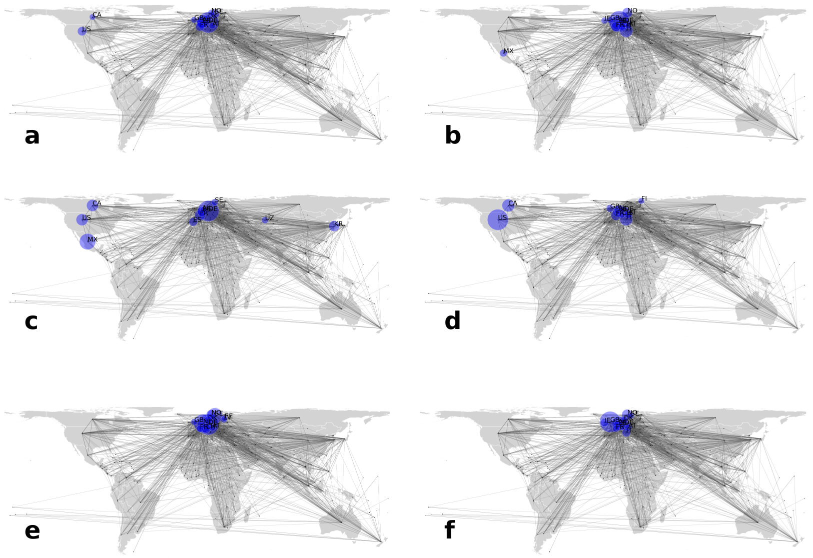

Figure SI. 4: Main importers (left panels) and exporters (right panels) for wastes of types I (a, b), II (c, d) and III (e, f).These values correspond to the in- and out-strengths (weighted degrees) of the corresponding countries in the respective W4 networks. Here, we highlight the first twelve importers and exporters. Country codes belong to the ISO-alpha-2 standard. Map tiles by Bjorn Sandvik, under CC BY-SA 3.0 available at http://thematicmapping.org/downloads/world_borders.php.

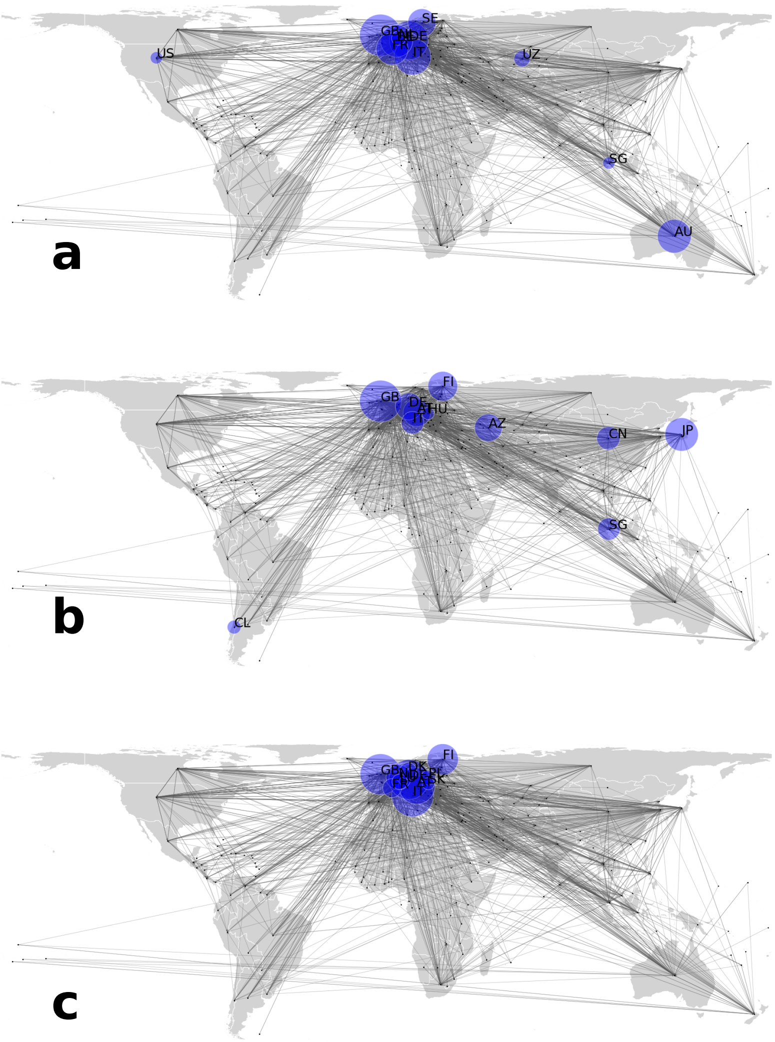

Figure SI. 5: Main countries to which more flux of wastes pass through them for types I (a), II (b) and III (c). These values correspond to the betweenness centrality of the corresponding countries in the respective W4 networks. Map tiles by Bjorn Sandvik, under CC BY-SA 3.0 available at http://thematicmapping.org/downloads/world_borders.php.

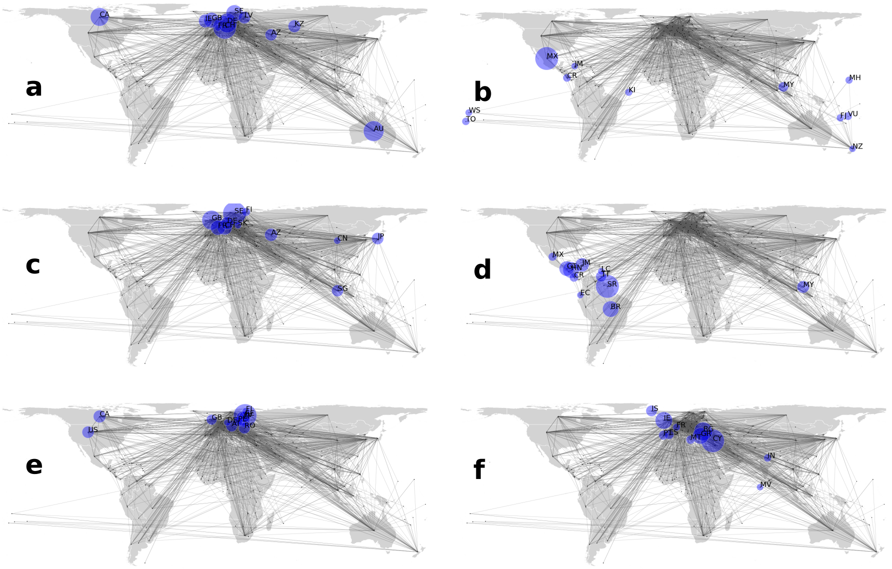

Figure SI. 6: Main countries to which the traffic of the network is closer from all other territories in the network. Values correspond to In-closeness and Out-closeness centrality for type I (a,b), II (c,d), II (e,f). Main countries which are closer to all countries/territories in the waste network for out-closeness centrality of type III (c). We highlight the first twelve hubs of each networks. Country codes belong to the ISO-alpha-2 standard. Map tiles by Bjorn Sandvik, under CC BY-SA 3.0 available at http://thematicmapping.org/downloads/world_borders.php.

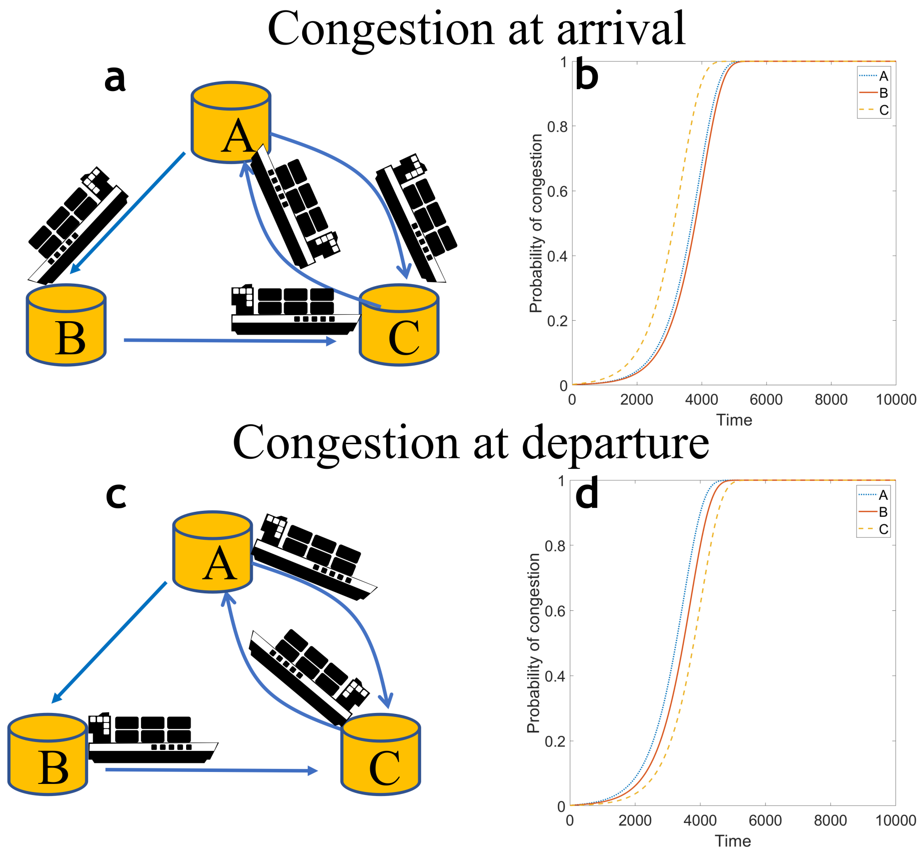

Figure SI. 7: Schematic illustration of the "congestion at arrival" (**a**) and "congestion at departure" (**c**) models and the time-evolution of the congestion propagation through the nodes using these models (**b** and **d**). Notice that in the congestion at arrival (panel **b**), node C reaches 50 % of congestion at a earlier time than A and B. In the congestion at departure (panel **d**), node A reaches 50 % of congestion earlier than B and C. Also notice that the ordering of congestion times at departure and arrival are not simply one the reverse of the other.

Theoretical modeling approach

The logistic dynamic model on a network is written as (see[39] for analysis in the case of Susceptible-Infected model, which is a particular case of the general model written here):

$$\frac{dw_i\left(t\right)}{dt} = \beta\left(1 - w_i\left(t\right)\right)\sum_{j=1}^{n} A_{ij}w_j\left(t\right), t \geq t_0, \tag{0.1}$$

where A_{ij} are the entries of the adjacency matrix of the W4 for the pair of countries i and j. In matrix-vector form it becomes:

$$\frac{d\mathbf{w}(t)}{dt} = \beta \left[I_N - \operatorname{diag}(\mathbf{w}(t))\right] A\mathbf{w}(t), \tag{0.2}$$

with initial condition $\mathbf{w}(0) = \mathbf{w}_0$,where I_N is the identity matrix of order N. This model can be rewritten as

$$\frac{1}{1 - w_i(t)} \frac{dw_i(t)}{dt} = \beta \sum_{j=1}^{n} A_{ij} \left(1 - e^{-(-\log(1 - w_j(t)))}\right), \tag{0.3}$$

which is equivalent to

$$\frac{dy_i(t)}{dt} = \beta \sum_{j=1}^{n} A_{ij} f(y_j(t)), \tag{0.4}$$

where $y_i(t) \coloneqq g(x_i(t)) = -\log(1 - w_i(t)) \in [0, \infty]$, $f(y) \coloneqq 1 - e^{-y} = g^{-1}(y)$.

We now consider a dynamics with non-locality by time or dynamic memory as described in the main text. To write the logistic model in this new context we start as follows. Let $0 < \alpha < 1$, then

$$\begin{cases} \displaystyle\int_0^t g_{1-\alpha}(t-\tau) \frac{(1-w_i)'(\tau)}{w_i(\tau)} d\tau = -\beta^\alpha (1-w_i)(t), \\ \displaystyle\int_0^t g_{1-\alpha}(t-\tau) \frac{w_i'(\tau)}{(1-w_i)(\tau)} d\tau = \beta^\alpha w_i(t). \end{cases}$$

Then, we have

$$\int_0^t g_{1-\alpha}(t-\tau) \frac{w_i'(\tau)}{1-w_i(\tau)} d\tau = \beta^\alpha w_i(t), \tag{0.5}$$

which for the case of a network is written as

$$\int_0^t g_{1-\alpha}(t-\tau) \frac{w_i'(\tau)}{1-w_i(\tau)} d\tau = \beta^\alpha \sum_{j=1}^{n} A_{ij} w_j, \quad t > 0, w_i(0) \in [0,1]. \tag{0.6}$$

We can rewrite (0.6) in a matrix-vector form:

$$D_t^\alpha(-\log(\mathbf{1} - \mathbf{w}))(t) = \beta^\alpha A\mathbf{w}(t), \tag{0.7}$$

with the logarithm taken entrywise, and with initial condition $\mathbf{w}(0) = \mathbf{w}_0$.

In order to solve analytically the previous equation we apply the Lee-Tenneti-Eun (LTE) transformation[40] which produces the following linearized equation

$$D_t^\alpha \hat{\mathbf{y}}(t) = \beta^\alpha A \operatorname{diag}(\mathbf{1} - \mathbf{w}_0) \hat{\mathbf{y}}(t) + \beta^\alpha A \mathbf{b}(\mathbf{w}_0), \tag{0.8}$$

where $\hat{\mathbf{w}}(t) = f(\hat{\mathbf{y}}(t))$ in which $\hat{\mathbf{w}}(t)$ is an approximate solution to the fractional SI model, $\hat{\mathbf{y}}$ is the solution of (0.8) with initial condition $\hat{\mathbf{y}}(0) = g(\mathbf{x}(0))$,

$\mathbf{1}$ is the all-ones vector, and $\mathbf{b}(\mathbf{w}) := \mathbf{w} + (\mathbf{1} - \mathbf{w})\log(\mathbf{1} - \mathbf{w})$. For convenience, we write $\Omega := \operatorname{diag}(\mathbf{1} - \mathbf{w}_0)$, and $\hat{A} = A\Omega$. Then, we have proved that this approximate solution $\hat{\mathbf{w}}(t)$ is a non-divergent upper bound to the exact solution $\mathbf{x}(t)$.

Theorem 1. *For any $t \geq 0$, we have*

$$\mathbf{w}(t) \preceq \hat{\mathbf{w}}(t) = f(\hat{\mathbf{y}}(t)),$$

under the same initial conditions $w_0 := w(0) = \hat{w}(0)$, where the solution $\hat{\mathbf{y}}$ of (0.8) is given by

$$\hat{\mathbf{y}}(t) = E_{\alpha,1}\left((\beta t)^{\alpha}\hat{A}\right)g(\mathbf{w}_0) + \sum_{n=0}^{\infty}\frac{(\beta t)^{\alpha(n+1)}\hat{A}^{n}A\mathbf{b}(\mathbf{w}_0)}{\Gamma(\alpha(n+1)+1)}. \tag{0.9}$$

Furthermore, $\|\hat{\mathbf{w}}(t) - \mathbf{w}(t)\| \to 0$ and $\|\tilde{\mathbf{w}}(t) - \mathbf{w}(t)\| \to \infty$ as t goes to infinity.

We also proved that when all values of the initial condition are smaller than one, i.e., $\mathbf{w}_0 \preceq \mathbf{1}$, which means that at the starting point of the simulation no country is completely congested of waste, the solution of the fractional logistic waste congestion model is

$$\hat{\mathbf{y}}(t) = g(\mathbf{w}_0) + \left[E_{\alpha,1}\left((\beta t)^{\alpha}\hat{A}\right) - I\right]\Omega^{-1}\mathbf{w}(0). \tag{0.10}$$

This is important because if we consider the plausible case that the probability of getting congested at $t = 0$ is the same for every country, which mathematically is written as: $w_0 = \frac{c}{N}$ where $c \in \mathbb{R}^{+}$, we have that

$$\hat{\mathbf{y}}(t) = \left(\frac{1-\gamma}{\gamma}\right)E_{\alpha,1}\left(t^{\alpha}\beta^{\alpha}\gamma A\right)\vec{\mathbf{1}} - \left(\frac{1-\gamma}{\gamma} + \log\gamma\right)\mathbf{1}, \tag{0.11}$$

where $\gamma = 1 - w_0$ and we have used the fact that $\operatorname{diag}(\mathbf{1} - \mathbf{w}(0)) = \gamma I$, where I is the identity matrix.

The Mittag-Leffler function $E_{\alpha,1}\left(\zeta A\right)$ with $\zeta = (\beta t)^{1/2}\gamma$, which appears in the approximate solution of the congestion models described in the main text, belongs to the class of matrix functions of the adjacency matrix.[41] It can be written as[42–45]

$$E_{\alpha,1}\left(\zeta A\right) = \sum_{k=0}^{\infty}\frac{(\zeta A)^{k}}{\Gamma(\alpha k+1)}, \alpha > 0. \tag{0.12}$$

If we expand the first terms of this matrix function for a pair of countries v and w we get:

$$\left(E_{\alpha,1}\left(\zeta A\right)\right)_{vw} = \frac{\zeta(A)_{vw}}{\Gamma(\alpha+1)} + \frac{\zeta^{2}(A^{2})_{vw}}{\Gamma(2\alpha+1)} + \frac{\zeta^{3}(A^{3})_{vw}}{\Gamma(3\alpha+1)} + \cdots. \tag{0.13}$$

The first term is different from zero only if the country v exports some amount of waste to country w. The second term accounts for the export of v to any country i, which then exports to w: $v \to i \to w$. The third term accounts for a chain of the type: $v \to i \to j \to w$ or of an interchange: $v \to w \to v \to w$. In every case the amounts exported from one country to another are taken into account. Notice that such chains could be of infinite length, but their importance is diminished by the denominator of each particular term, given by the Euler gamma functions.

A centrality index, like the strengths (in- and out-) only take into account the contribution of exports/import between pairs of connected nodes in the network. In case that the country v exports some amount of waste to country w, the out-strength of v is given by the first term of the previous series expansion. However, this strength measures do not take into account the chains of lengths longer than one, such as $v \to i \to w$, or $v \to i \to j \to w$. More importantly, if two countries v and w are not connected in the network, the strengths measures fail to account for possible indirect exports/imports between these two countries through an intermediary, such as in $v \to i \to w$.

This lack of correlation between the first order measures, like strength, and higher order ones are revealed by the plots (see Fig. SI. 8) of the ratio of both strength measures and the index of risk of waste congestion defined here.

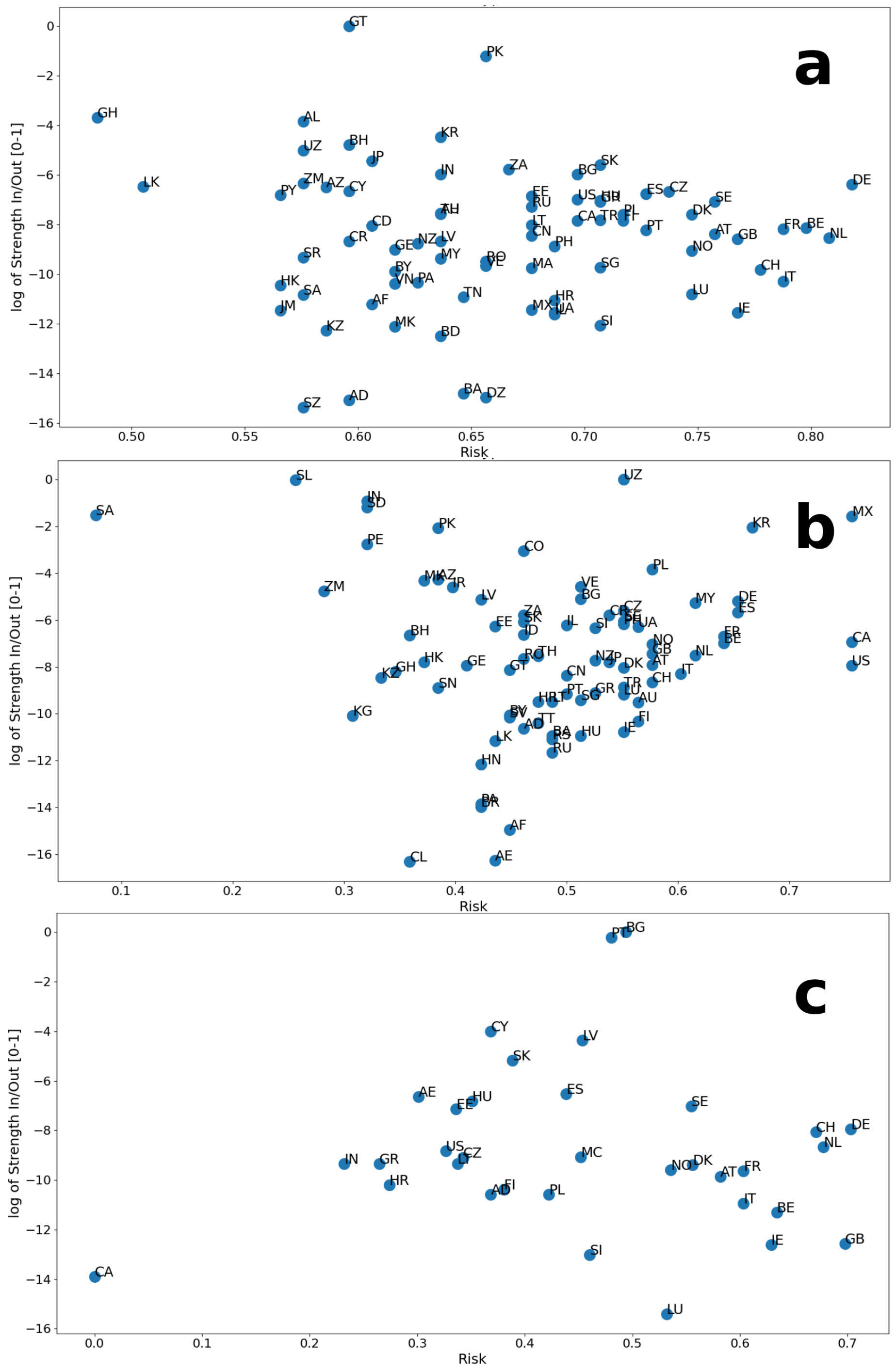

Figure SI. 8: Scatter plot between a local measure (strength-in/strength-out), vs congestion risk for countries involved in networks of types I (**a**) with Pearson index of 0.01, II (**b**) with Pearson index of 0.00, and III (**c**).with Pearson index of 0.10 .

Chemical fingerprints

country	Evidence of CF found	Ref.
AF	Reports from foreign military bases on emissions of VOC, PAH, and HM; 77% of the volume of waste generated in Kabul are uncollectedmeaning 1180 m^3 of waste (830 tons) uncollected.	46–48
BD	Heavy metal contamination of fruits, vegetables, fish and ther foodstuffs with risk for human health; serious mismanagement of medical waste in the capital with serious risk of human health; Heavy pollution with POP, particularly polychlorinated byphenyls (PCB).	49–56
BJ	The Benin Republic has the highest per capita hazardous waste generation in Africa at 65 kg/person/annum; Large amounts of e-waste reported; 1,698 tons of HW incinerated, none recycled nor landfilled; large exposure of inhabitants of the capital to BTEX.	57, 58
BF	Heavy metals contamination of soils from informal settlements, peri-urban agriculture and unregulated waste dumping; problems with waste management producing heavy metals contamination and with impact on human health.	59–62
CN	Heavy metals, VOC and POP pollution due to e-waste disposal and mismanagement with serious thread for human health; high contamination levels of PCB from equipments; emission and speciation of VOC from anthropogenic sources, ncluding high levels of BTEX; high levels of pollution from medical wastes and their incineration.	63–75
CD	Heavy metals and POP contamination in river, estuary, and marine sediments from Atlantic Coast; impact of heavy metals on human health in children and adult populations; contamination of water resources and food chain by POP.	76–79
DJ	Reports of a shipment of containers with up to 20 metric tons of toxic chemicals were found leaking in the port of Djibouti with potential pollution by Arsenic.	80

ET	Problems with uncontrolled waste disposal and heavy metals contamination of soils and waters in Addis Ababa; contamination by VOC in urban environment; high levels of pollution by POP, specially PCB and dioxins at different lcatins and levels of the trophic chain; seriuos problems of mismanagement of medical wastes with reported cases of hepatitis B and C directly related to them.	81–91
GN	Heavy metals contamination in the Gulf of Guinea.	92, 93
IN	Heavy metals contamination due to diverse wastes, including e-waste, with serious thread for human health; high levels of VOC from waste dumps, including high levels of BTEX; problems with dumping and informal recycling of medical waste with important human health problems.	94–105
LS	Open waste combustion emissions of CO2 are estimated to be more than each country's total national CO2 emissions as reported by the United Nations; As and Pb concentration levels found in *Cyprinus carpio* were higher than the WHO permissible limits recommended for fish consumption Maqalika Reservoir –Maseru, Lesotho; High levels of disposed e-waste are reported.	106–108
LR	Waste management activities are getting worse daily due to shortage of a comprehensive waste management framework; reports of mismanagement of healthcare waste.	109, 110
MG	High levels of HW landfilled (33,812 tons), none recycled and 12,145 tons incinerated; reports of illegal import of car batteries and extraction of Pb for export.	57, 111
MR	Contamination by POP like PAHs in Atlantic coast of Mauritania (Levrier Bay Zone); high levels of PCB contamination in marine ecosystems front of the coast of Mauritania.	112–114
MX	High levels of BTEX found due among other causes to waste burning; exposure of children to mixtures of pollutants including PAH, PCB and HM (As and Pb) in a site with a hazardous waste landfill; reported levels of emission factors for polychlorinated and polybrominated dibenzodioxins/dibenzofurans and polybrominated diphenyl ethers from open burning of domestic waste; environmental pollution due to e-waste	115–122

MA	Only 37% of the collected SW is disposed off in controlled landfills; non-civic behaviors and deterioration of the environment have been reported; BTEX, PAH and PCB are reported in different environmental reservoirs.	123–127
MZ	Heavy metals and organic chemicals, including pharmaceuticals, in soils and waters.	128–130
NG	Heavy metals contamination and ecological risks from municipal central dumpsite; contamination by heavy metals, VOC and POP due to informal e-waste recycling; PAHs and PCB in groundwater around waste dumpsites in South-West Nigeria.	131–137
PK	Heavy metals pollution from diverse waste sources, including e-waste, recognized as an emerging problem and medical waste incineration; high levels of VOC, including BTEX in urban atmosphere; public health problems from hospital solid waste mismanagement.	138–145
PG	Heavy metal water pollution in Depapre waters.	146
SN	Dramatic problems with household waste collection; reported waste contamination at different reservoirs by HM, mainly in the coast; environmental issues with biomedical waste disposal; 18 children died from a rapidly progressive central nervous system disease of unexplained origin in a community involved in the recycling of used lead-acid batteries; high levels of contamination with Pb in homes and soil in surrounding areas dedicated to car battery informal recycling, several children showed severe neurologic features of toxicity; high levels of VOC contamination at different environmental places.	147–155
SL	Heavy metals and POP contamination due to e-waste recycling; high levels of exposition to dioxins and furans; mismanagement of solid waste depositions with environmental and human health risk.	156–158
UZ	Pollution by heavy metals with impact on human health; contamination of soils with POP, particularly by PAHs.	159–161

Countries/territories without EPI

Type I			Type II			Type III	
country	R_i		country	R_i		country	R_i
San Marino	0.646		Andorra	0.461		Monaco	0.452
Liechtenstein	0.616		Puerto Rico	0.461		San Marino	0.422
Monaco	0.616		Faroe Islands	0.423		Andorra	0.368
Andorra	0.596		San Marino	0.397		Faroe Islands	0.226
Guernsey	0.586		Monaco	0.385			
Isle of Man	0.586		Hong Kong	0.372			
Hong Kong	0.566		Liechtenstein	0.372			
Faroe Islands	0.555		New Caledonia	0.359			
New Caledonia	0.545		Guernsey	0.446			
Falkland Islands	0.515		Isle of Man	0.333			
St. Barthelemy	0.495		Jersey	0.333			
Jersey	0.485		Guernsey	0.308			
Palestine	0.384		Niue	0.308			
St. Kitts & Nevis	0.364		Cook Islands	0.256			
Cook Islands	0.333		Falkland Islands	0.243			
Niue	0.323		Palestine	0.159			
Tuvalu	0.313						

Table SI. 12: Values of the risk of congestion R_i of waste types I-III for countries/territories for which EPI are not reported. Therefore, we cannot built the PEIWC for these countries/territories although they can be at relatively HRIHDW.

Waste categories in types IV-VII

Waste of types IV-VII represents less than 0.001% of the total volume of waste traded in the world in the period 2003-2009. However, in volume it still represents 2866.79 tonnes of waste traded across the world: 821.92 tonnes (type IV), 295.90 tonnes (type V), 756.15 tonnes (type VI) and 992.83 tonnes (type VII). The different categories of the Basel Convention included in these four types are described in Tables SI. 13, SI. 14, SI. 15, and SI. 16, respectively.

category	description
A1010	Metal wastes and waste consisting of alloys of any of the following: Antimony, Arsenic, Beryllium, Cadmium, Lead, Mercury, Selenium, Tellurium, Thallium.
A1020	Waste having as constituents or contaminants, excluding metal waste in massive form, any of the following: Antimony; antimony compounds, Beryllium; beryllium compounds, Cadmium; cadmium compounds, Lead; lead compounds, Selenium; selenium compounds, Tellurium; tellurium compounds
A1030	Wastes having as constituents or contaminants any of the following: Arsenic; arsenic compounds, Mercury; mercury compounds, Thallium; thallium compounds
A1040	Wastes having as constituents any of the following: Metal carbonyls, hexavalent chromium compounds
A1050	Galvanic sludges
A1060	Waste liquors from the pickling of metals
A1070	Leaching residues from zinc processing, dust and sludges, such as jarosite, hematite, etc.
A1080	Waste zinc residues, containing lead and cadmium in concentrations sufficient to exhibit Annex III characteristics
A1090	Ashes from the incineration of insulated copper wire
A1100	Dusts and residues from gas cleaning systems of copper smelters
A1110	Spent electrolytic solutions from copper electrorefining and electrowinning operations
A1120	Waste sludges, excluding anode slimes, from electrolyte purification systems in copper electrorefining and electrowinning operations
A1130	Spent etching solutions containing dissolved copper
A1140	Waste cupric chloride and copper cyanide catalysts
A1150	Precious metal ash from incineration of printed circuit boards
A1160	Waste lead-acid batteries, whole or crushed
A1170	Unsorted waste batteries. Waste batteries containing Annex I constituents to an extent to render them hazardous
A1180	Waste electrical and electronic assemblies or scrap containing components such as accumulators and other batteries included on list A, mercury-switches, glass from cathode-ray tubes and other activated glass and PCB capacitors, or contaminated with Annex I constituents (e.g., cadmium, mercury, lead, polychlorinated biphenyl) to an extent that they possess any of the characteristics contained in Annex III
A1190	Waste metal cables coated or insulated with plastics containing or contaminated with coal tar, PCB, lead, cadmium, other organohalogen compounds or other Annex I constituents to an extent that they exhibit Annex III characteristics.

Table SI. 13: Waste categories included in the Basel Convention which are grouped in the type IV of wastes.

category	description
A2010	Glass waste from cathode-ray tubes and other activated glasses
A2020	Waste inorganic fluorine compounds in the form of liquids or sludges
A2030	Waste catalysts
A2040	Waste gypsum arising from chemical industry processes, when containing Annex I constituents to the extent that it exhibits an Annex III hazardous characteristic
A2050	Waste asbestos (dusts and fibres)
A2060	Coal-fired power plant fly-ash containing Annex I substances in concentrations sufficient to exhibit Annex III characteristics

Table SI. 14: Waste categories included in the Basel Convention which are grouped in the type V of wastes.

category	description
A3010	Waste from the production or processing of petroleum coke and bitumen
A3020	Waste mineral oils unfit for their originally intended use
A3030	Wastes that contain, consist of or are contaminated with leaded anti-knock compound sludges
A3040	Waste thermal (heat transfer) fluids
A3050	Wastes from production, formulation and use of resins, latex, plasticizers, glues/adhesives
A3060	Waste nitrocellulose
A3070	Waste phenols, phenol compounds including chlorophenol in the form of liquids or sludges
A3080	Waste ethers
A3090	Waste leather dust, ash, sludges and flours when containing hexavalent chromium compounds or biocides
A3100	Waste paring and other waste of leather or of composition leather not suitable for the manufacture of leather articles containing hexavalent chromium compounds or biocides
A3110	Fellmongery wastes containing hexavalent chromium compounds or biocides or infectious substances
A3120	Fluff-light fraction from shredding
A3130	Waste organic phosphorous compounds
A3140	Waste non-halogenated organic solvents
A3150	Waste halogenated organic solvents
A3160	Waste halogenated or unhalogenated non-aqueous distillation residues arising from organic solvent recovery operations
A3170	Wastes arising from the production of aliphatic halogenated hydrocarbons (such as chloromethane, dichloro-ethane, vinyl chloride, vinylidene chloride, allyl chloride and epichlorhydrin)
A3180	Wastes, substances and articles containing, consisting of or contaminated with polychlorinated biphenyl (PCB), polychlorinated<br>terphenyl (PCT), polychlorinated naphthalene (PCN) or Polybrominated biphenyl (PBB), or any other polybrominated analogues of these compounds, at a concentration level of 50 mg/kg or more
A3190	Waste tarry residues (excluding asphalt cements) arising from refining, distillation and any pyrolitic treatment of organic materials
A3200	Bituminous material (asphalt waste) from road construction and maintenance, containing tar

Table SI. 15: Waste categories included in the Basel Convention which are grouped in the type VI of wastes.

category	description
A4010	Wastes from the production, preparation and use of pharmaceutical products
A4020	Clinical and related wastes; that is wastes arising from medical, nursing, dental, veterinary, or similar practices, and wastes generated in hospitals or other facilities during the investigation or treatment of patients, or research projects
A4030	Wastes from the production, formulation and use of biocides and phytopharmaceuticals, including waste pesticides and herbicides which are off-specification, outdated, or unfit for their originally intended use
A4040	Wastes from the manufacture, formulation and use of wood preserving chemicals
A4050	Wastes that contain, consist of or are contaminated with any of the following: Inorganic cyanides, excepting precious-metal-bearing, residues in solid form containing traces of inorganic cyanides, organic cyanides
A4060	Waste oils/water, hydrocarbons/water mixtures, emulsions
A4070	Wastes from the production, formulation and use of inks, dyes, pigments, paints, lacquers, varnish
A4080	Wastes of an explosive nature
A4090	Waste acidic or basic solutions
A4100	Wastes from industrial pollution control devices for cleaning of industrial off-gases
A4110	Wastes that contain, consist of or are contaminated with any of the following: Any congenor of polychlorinated dibenzo-furan; Any congenor of polychlorinated dibenzo-p-dioxin
A4120	Wastes that contain, consist of or are contaminated with peroxides
A4130	Waste packages and containers containing Annex I substances in concentrations sufficient to exhibit Annex III hazard characteristics
A4140	Waste consisting of or containing off specification or outdated chemicals corresponding to Annex I categories and exhibiting Annex III hazard characteristics
A4150	Waste chemical substances arising from research and development or teaching activities which are not identified and/or are new and whose effects on human health and/or the environment are not known
A4160	Spent activated carbon

Table SI. 16: Waste categories included in the Basel Convention which are grouped in the type VII of wastes.

PEIWS analysis of wastes types IV-VII

Following the same procedure described in Methods we build the PEIWS of the four types of waste IV-VII, which are illustrated in Fig. SI. 9. Using the same approach as for the waste types I-III we identify these countries at HRIHDW. In total in the four types of waste there are 29 countries at HRIHDW, 22 of which coincide with countries previously identified at HRIHDW for waste types I-III. The new countries at HRIHDW, i.e., not identified for types I-III, are Kazakhstan, Mongolia, Côte d'Ivoire, Saudi Arabia, Tanzania, Kenya and Oman. Wastes of types IV and VI are the ones with the largest number of countries at HRIHDW with 15 and 12, respectively, while types V and VII have 8 and 9 countries at HRIHDW, respectively. By continents, Africa is again the one having more countries at HRIHDW with 12, followed by Asia (9) and then Middle East and Europe with 4 each.

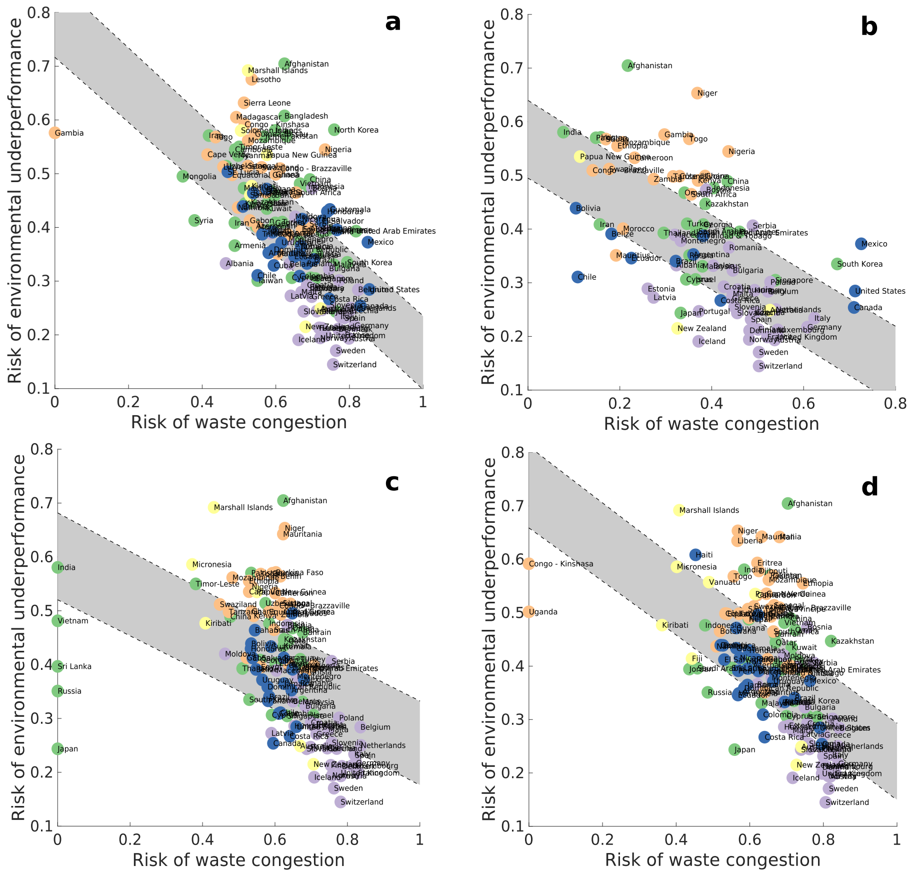

Figure SI. 9: **PEIWC of wastes types IV-VII**. (a)-(d) Illustration of the PEIWC for wastes of types IV-VII, respectively. The risks of waste congestion are calculated from the simulated dynamics using a fractional logistic model described in Methods. The index of risk of environmental underperformance is obtained from the Yale University environmental performance index (EPI). Nodes are colored by the continent to which thecountry belongs to: blue (Americas), purple (Europe), yellow (Africa), green (Asia)

References

[1] Brusseau ML, Artiola JF. Chemical contaminants. In Environmental and Pollution Science (Academic Press, 175-190 2019).

[2] Weiss FT, Leuzinger M, Zurbrügg C, Eggen HI. Chemical pollution in low- and middle-income countries. (Swiss Federal Institute of Aquatic Science and Technology Press, Switzerland 2016).

[3] Fazzo L, Minichilli F, Santoro M, Ceccarini A, Della Seta M, Bianchi F, Comba P, Martuzzi M. Hazardous waste and health impact: A systematic review of the scientific literature. Environmental health. 16(1) 1-1 (2017).

[4] Ogunseitan OA, Schoenung JM, Saphores JD, Shapiro AA. The electronics revolution: From e-wonderland to e-wasteland. Science. 326(5953), 670-1 (2009).

[5] Hossain MS, Santhanam A, Norulaini NN, Omar AM. Clinical solid waste management practices and its impact on human health and environment–A review. Waste management. 31(4), 754-66 (2011).

[6] Harhay MO, Halpern SD, Harhay JS, Olliaro PL. Health care waste management: A neglected and growing public health problem worldwide. Tropical Medicine & International Health. 14(11), 1414-7 (2009).

[7] Babanyara YY, Ibrahim DB, Garba T, Bogoro AG, Abubakar MY. Poor Medical Waste Management (MWM) practices and its risks to human health and the environment: A literature review. Int J Environ Ealth Sci Eng. 11(7), 1-8 (2013).

[8] Chen DM, Bodirsky BL, Krueger T, Mishra A, Popp A. The world's growing municipal solid waste: Trends and impacts. Environmental Research Letters. 15(7), 074021 (2020).

[9] Thomas JK, Fannin D. The transboundary trade of hazardous wastes. Environmental Justice. 4(1), 55-62 (2011).

[10] Clapp J. Toxic Exports (Cornell University Press, 2018).

[11] Marin G, Nicolli F, Zecca E. Drivers of international shipments of hazardous waste: The role of policy and technology endowment. SEEDS (Sustainability Environmental Economics and Dynamics Studies, Italy. 2017).

[12] Sonak S, Sonak M, Giriyan A. Shipping hazardous waste: implications for economically developing countries. International environmental agreements: Politics, law and economics. 8(2), 143-59 (2008).

[13] Walters R, Fuentes M.A. Waste Crime and the Global Transference of Hazardous Substances: A Southern Green Perspective. Critical Criminology. 28, 463–480 (2020).

[14] Fayiga AO, Ipinmoroti MO, Chirenje T. Environmental pollution in Africa. Environment, Development and Sustainability. 20, 41-73 (2018).

[15] Akpan VE, Olukanni DO. Hazardous waste Management: An African overview. Recycling. 5(3), 15 (2020).

[16] Udofia EA, Nriagu J. Health-care waste in Africa: A silent crises. Global Health Perspect. 1(1), 3-10 (2013).

[17] Udofia EA, Fobil JN, Gulis G. Solid medical waste management in Africa. African journal of Environmental Science and Technology. 9(3), 244-54 (2015).

[18] Orisakwe OE, Frazzoli C, Ilo CE, Oritsemuelebi B. Public health burden of e-waste in Africa. Journal of Health and Pollution. 9(22), 190610 (2019).

[19] Ishchenko VA. Environment contamination with heavy metals contained in waste. Environmental Problems. 3(1), 21-24. (2018).

[20] Alloway BJ. Heavy metals in soils: trace metals and metalloids in soils and their bioavailability (Springer Science & Business Media 2012).

[21] Purchase D, Abbasi G, Bisschop L, Chatterjee D, Ekberg C, Ermolin M, Fedotov P, Garelick H, Isimekhai K, Kandile NG, Lundström M. Global occurrence, chemical properties, and ecological impacts of e-wastes (IUPAC Technical Report). Pure and Applied Chemistry. 92(11), 1733-67 (2020).

[22] Hossain MS, Santhanam A, Norulaini NN, Omar AM. Clinical solid waste management practices and its impact on human health and environment–A review. Waste management. 31(4), 754-66 (2011).

[23] Holm O, Hansen E, Lassen C, Stuer-Lauridsen F, Jesper K. Heavy Metals in Waste–Final Report (European Commission DG ENV. E3, Project ENV.E.3/ETU/2000/0058, 2002).

[24] Ishchenko V, Vasylkivskyi I. Environmental pollution with heavy metals: Case study of the Household Waste. in: Sustainable Production: Novel Trends in Energy, Environment and Material Systems (Springer, Cham, 2020).

[25] Siddique S, Kwoffie L, Addae-Afoakwa K, Yates K, Njuguna J. Oil based drilling fluid waste: An overview on environmentally persistent pollutants. in: IOP Conference Series: Materials Science and Engineering (IOP Publishing, 195, 012008 2017).

[26] Islam MS, Ahmed MK, Raknuzzaman M, Habibullah-Al-Mamun M, Kundu GK. Heavy metals in the industrial sludge and their ecological risk: A case study for a developing country. Journal of Geochemical Exploration. 172, 41-9 (2017).

[27] He C, Cheng J, Zhang X, Douthwaite M, Pattisson S, Hao Z. Recent advances in the catalytic oxidation of volatile organic compounds: A review based on pollutant sorts and sources. Chemical reviews. 119(7), 4471-568 (2019).

[28] He Z, Li G, Chen J, Huang Y, An T, Zhang C. Pollution characteristics and health risk assessment of volatile organic compounds emitted from different plastic solid waste recycling workshops. Environment international. 77, 85-94 (2015).

[29] Niu Z, Kong S, Zheng H, Yan Q, Liu J, Feng Y, Wu J, Zheng S, Zeng X, Yao L, Zhang Y. Temperature dependence of source profiles for volatile organic compounds from typical volatile emission sources. Science of The Total Environment. 751, 141741 (2021).

[30] Sabel GV, Clark TP. Volatile organic compounds as indicators of municipal solid waste leachate contamination. Waste Management & Research. 2(2), 119-30 (1984).

[31] Liu R, Chen J, Li G, An T. Using an integrated decontamination technique to remove VOCs and attenuate health risks from an e-waste dismantling workshop. Chemical Engineering Journal. 318, 57-63 (2017).

[32] Chen D, Liu R, Lin Q, Ma S, Li G, Yu Y, Zhang C, An T. Volatile organic compounds in an e-waste dismantling region: From spatial-seasonal variation to human health impact. Chemosphere. 275, 130022 (2021).

[33] Carlsen L, Bruggemann R, Kenessov B. Use of partial order in environmental pollution studies demonstrated by urban BTEX air pollution in 20 major cities worldwide. Science of the Total Environment. 610, 234-43 (2018).

[34] Fiedler H. Release inventories of polychlorinated dibenzo-p-dioxins and polychlorinated dibenzofurans. in: Dioxin and Related Compounds (Springer, Cham, 1-27, 2015).

[35] Kanan S, Samara F. Dioxins and furans: A review from chemical and environmental perspectives. Trends in Environmental Analytical Chemistry. 17, 1-3 (2018).

[36] Nowak P, Kucharska K, Kamiński M. Ecological and health effects of lubricant oils emitted into the environment. International journal of environmental research and public health. 16(16), 3002 (2019).

[37] UNEP. Polychlorinated Biphenyls (PCB) Inventory Guidance (UN Economic Division, 2016).

[38] Liu J, Tan Y, Song E, Song Y. A critical review of Polychlorinated Biphenyls metabolism, metabolites, and their correlation with oxidative stress. Chemical Research in Toxicology. 33(8), 2022-42 (2020).

[39] W. Mei, S. Mohagheghi, S. Zampieri, F. Bullo, On the dynamics of deterministic epidemic propagation over networks. Annual Reviews in Control. 44, 116–128 (2017).

[40] C.-H. Lee, S. Tenneti, D. Y. Eun, Transient dynamics of epidemic spreading and its mitigation on large networks, in: Proceedings of the Twentieth ACM International Symposium on Mobile Ad Hoc Networking and Computing, Mobihoc '19 (Association for Computing Machinery, New York, NY, USA, 191200, 2019).

[41] Estrada E, Hatano N. Communicability in complex networks. Physical Review E. 77(3), 036111 (2008).

[42] Matychyn I. On Computation of Matrix Mittag-Leffler Function. arXiv preprint arXiv:1706.01538, (2017).

[43] Garrappa R, Popolizio M. Computing the matrix Mittag-Leffler function with applications to fractional calculus. Journal of Scientific Computing. 77, 129–153 (2018).

[44] Sadeghi A, Cardoso JR. Some notes on properties of the matrix Mittag-Leffler function. Applied Mathematics and Computation. 338, 733-8 (2018).

[45] Fulger D, Scalas E, Germano G. Monte Carlo simulation of uncoupled continuous-time random walks yielding a stochastic solution of the space-time fractional diffusion equation. Physical Review E. 77(2), 021122 (2008).

[46] Woodall, Brian D., et al. Emissions from small-scale burns of simulated deployed US military waste. Environmental science & technology 46.20 (2012): 10997-11003.

[47] Magnusson, Roger, Lars Hägglund, and Håkan Wingfors. Broad exposure screening of air pollutants in the occupational environment of Swedish soldiers deployed in Afghanistan. Military medicine 177.3 (2012): 318-325.

[48] Glawe, Ulrich, Chettiyappan Visvanathan, and Mohammed Alamgir. Solid waste management in least developed Asian countries–a comparative analysis. International Conference on Integrated Solid Waste Management in Southeast Asian Cities. 2005.

[49] Ahmed MK, Baki MA, Kundu GK, Islam MS, Islam MM, Hossain MM. Human health risks from heavy metals in fish of Buriganga river, Bangladesh. SpringerPlus. 2016 Dec;5(1):1-2.

[50] Shaheen N, Irfan NM, Khan IN, Islam S, Islam MS, Ahmed MK. Presence of heavy metals in fruits and vegetables: Health risk implications in Bangladesh. Chemosphere. 2016 Jun 1;152:431-8.

[51] Saha N, Zaman MR. Evaluation of possible health risks of heavy metals by consumption of foodstuffs available in the central market of Rajshahi City, Bangladesh. Environmental monitoring and assessment. 2013 May;185(5):3867-78.

[52] Hassan MM, Ahmed SA, Rahman KA, Biswas TK. Pattern of medical waste management: existing scenario in Dhaka City, Bangladesh. BMC public health. 2008 Dec;8(1):1-0.

[53] Syed EH, Mutahara M, Rahman M. Medical waste management (MWM) in Dhaka, Bangladesh: it'sa review. Home Health Care Management & Practice. 2012 Jun;24(3):140-5.

[54] Nøst TH, Halse AK, Randall S, Borgen AR, Schlabach M, Paul A, Rahman A, Breivik K. High concentrations of organic contaminants in air from ship breaking activities in Chittagong, Bangladesh. Environmental science & technology. 2015 Oct 6;49(19):11372-80.

[55] Habibullah-Al-Mamun M, Ahmed MK, Islam MS, Tokumura M, Masunaga S. Occurrence, distribution and possible sources of polychlorinated biphenyls (PCBs) in the surface water from the Bay of Bengal coast of Bangladesh. Ecotoxicology and environmental safety. 2019 Jan 15;167:450-8.

[56] Habibullah-Al-Mamun M, Ahmed MK, Islam MS, Hossain A, Tokumura M, Masunaga S. Polychlorinated biphenyls (PCBs) in commonly consumed seafood from the coastal area of Bangladesh: occurrence, distribution, and human health implications. Environmental Science and Pollution Research. 2019 Jan;26(2):1355-69.

[57] Akpan, Victor E., and David O. Olukanni. Hazardous waste Management: an African overview. Recycling 5.3 (2020): 15.

[58] Tohon, Honesty Gbèdolo, et al. BTEX air concentrations and self-reported common health problems in gasoline sellers from Cotonou, Benin. International journal of environmental health research 25.2 (2015): 149-161.

[59] Sako A, Sawadogo S, Nimi M, Ouédraogo M. Hydrogeochemical and pollution characterization of a shallow glauconitic sandstone aquifer in a periurban setting of Bobo-Dioulasso, southwestern Burkina Faso. Environmental Earth Sciences. 2020 Jun;79:1-8.

[60] Patricia K, Bila Gérard S, Jean Fidèle N, Jean K. Environmental Impacts of Waste Management Deficiencies and Health Issues: A Case Study in the City of Kaya, Burkina Faso. Journal of environmental protection. 2013 Sep 30;2013.

[61] Yiougo LS, Oyedotun TD, Some CY, Da EC. Urban cities and waste generation in developing countries: A gis evaluation of two cities in burkina faso. Journal of Urban and Environmental Engineering. 2013 Jul 1;7(2):280-5.

[62] Fregonese F, Siekmans K, Kouanda S, Druetz T, Ly A, Diabaté S, Haddad S. Impact of contaminated household environment on stunting in children aged 12–59 months in Burkina Faso. J Epidemiol Community Health. 2017 Apr 1;71(4):356-63.

[63] Li W, Achal V. Environmental and health impacts due to e-waste disposal in China–A review. Science of The Total Environment. 2020 Oct 1;737:139745.

[64] Song Q, Li J. A review on human health consequences of metals exposure to e-waste in China. Environmental Pollution. 2015 Jan 1;196:450-61.

[65] Wong CS, Duzgoren-Aydin NS, Aydin A, Wong MH. Evidence of excessive releases of metals from primitive e-waste processing in Guiyu, China. Environmental Pollution. 2007 Jul 1;148(1):62-72.

[66] Guo Y, Huang C, Zhang H, Dong Q. Heavy metal contamination from electronic waste recycling at Guiyu, Southeastern China. Journal of environmental quality. 2009 Jul;38(4):1617-26.

[67] Xing Y, Lu Y, Dawson RW, Shi Y, Zhang H, Wang T, Liu W, Ren H. A spatial temporal assessment of pollution from PCBs in China. Chemosphere. 2005 Aug 1;60(6):731-9.

[68] Cai QY, Mo CH, Wu QT, Katsoyiannis A, Zeng QY. The status of soil contamination by semivolatile organic chemicals (SVOCs) in China: a review. Science of the Total Environment. 2008 Jan 25;389(2-3):209-24.

[69] Wei W, Wang S, Chatani S, Klimont Z, Cofala J, Hao J. Emission and speciation of non-methane volatile organic compounds from anthropogenic sources in China. Atmospheric Environment. 2008 Jun 1;42(20):4976-88.

[70] Chan LY, Chu KW, Zou SC, Chan CY, Wang XM, Barletta B, Blake DR, Guo H, Tsai WY. Characteristics of nonmethane hydrocarbons (NMHCs) in industrial, industrial-urban, and industrial-suburban atmospheres of the Pearl River Delta (PRD) region of south China. Journal of geophysical research: atmospheres. 2006 Jun 16;111(D11).

[71] Du Z, Mo J, Zhang Y, Xu Q. Benzene, toluene and xylenes in newly renovated homes and associated health risk in Guangzhou, China. Building and Environment. 2014 Feb 1;72:75-81.

[72] Yang L, Zhou Y, Shi B, Meng J, He B, Yang H, Yoon SJ, Kim T, Kwon BO, Khim JS, Wang T. Anthropogenic impacts on the contamination of pharmaceuticals and personal care products (PPCPs) in the coastal environments of the Yellow and Bohai seas. Environment international. 2020 Feb 1;135:105306.

[73] Yong Z, Gang X, Guanxing W, Tao Z, Dawei J. Medical waste management in China: A case study of Nanjing. Waste management. 2009 Apr 1;29(4):1376-82.

[74] Yan M, Li XD, Lu SY, Chen T, Chi Y, Yan JH. Persistent organic pollutant emissions from medical waste incinerators in China. Journal of Material Cycles and Waste Management. 2011 Oct;13(3):213-8.

[75] Wei Y, Cui M, Ye Z, Guo Q. Environmental challenges from the increasing medical waste since SARS outbreak. Journal of cleaner production. 2021 Apr 1;291:125246.

[76] Suami RB, Sivalingam P, Al Salah DM, Grandjean D, Mulaji CK, Mpiana PT, Breider F, Otamonga JP, Poté J. Heavy metals and persistent organic pollutants contamination in river, estuary, and marine sediments from Atlantic Coast of Democratic Republic of the Congo. Environmental Science and Pollution Research. 2020 Jun;27(16):20000-13.

[77] Bora BK, Ramos-Crawford AL, Sikorskii A, Boivin MJ, Lez DM, Mumba-Ngoyi D, Mukalay AM, Okitundu-Luwa D, Tshala-Katumbay D. Concurrent exposure to heavy metals and cognition in school-age children in Congo-Kinshasa: a complex overdue research agenda. Brain research bulletin. 2019 Feb 1;145:81-6.

[78] Kabamba M, Basosila N, Mulaji C, Mata H, Tuakuila J. Toxic heavy metals in ambient air of Kinshasa, Democratic Republic Congo. J Environ Anal Chem. 2016;3(178):2.

[79] Ngweme GN, Al Salah DM, Laffite A, Sivalingam P, Grandjean D, Konde JN, Mulaji CK, Breider F, Poté J. Occurrence of organic micropollutants and human health risk assessment based on consumption of Amaranthus viridis, Kinshasa in the Democratic Republic of the Congo. Science of The Total Environment. 2021 Feb 1;754:142175.

[80] Chou, C. H., and Christopher T. De Rosa. Case studies--arsenic. International Journal of Hygiene and Environmental Health 206.4-5 (2003): 381-386.

[81] Alemayehu T. The impact of uncontrolled waste disposal on surface water quality in Addis Ababa, Ethiopia. SINET: Ethiopian Journal of Science. 2001;24(1):93-104.

[82] Alemayehu T. Heavy metal concentration in the urban environment of Addis Ababa, Ethiopia. Soil & Sediment Contamination. 2006 Dec 1;15(6):591-602.

[83] Do DH, Van Langenhove H, Walgraeve C, Hayleeyesus SF, De Wispelaere P, Dewulf J, Demeestere K. Volatile organic compounds in an urban environment: a comparison among Belgium, Vietnam and Ethiopia. International Journal of Environmental Analytical Chemistry. 2013 Mar 1;93(3):298-314.

[84] Kassegne AB, Okonkwo JO, Berhanu T, Daso AP, Olukunle OI, Asfaw SL. Ecological risk assessment of organochlorine pesticides and polychlorinated biphenyls in water and surface sediment samples from Akaki River catchment, central Ethiopia. Emerging Contaminants. 2020 Jan 1;6:396-404.

[85] Deribe E, Rosseland BO, Borgstrøm R, Salbu B, Gebremariam Z, Dadebo E, Norli HR, Eklo OM. Bioaccumulation of persistent organic pollutants (POPs) in fish species from Lake Koka, Ethiopia: the influence of lipid content and trophic position. Science of the total environment. 2011 Dec 1;410:136-45.

[86] Dirbaba NB, Li S, Wu H, Yan X, Wang J. Organochlorine pesticides, polybrominated diphenyl ethers and polychlorinated biphenyls in surficial sediments of the Awash River Basin, Ethiopia. PloS one. 2018 Oct 4;13(10):e0205026.

[87] Urbaniak M, Zalewski M. Polychlorinated Dibenzo-p-Dioxins and Polychlorinated Dibenzofurans in Sediments from Two Ethiopian Rift Valley Lakes. Polish Journal of Environmental Studies. 2011 Jul 1;20(4).

[88] Mato RR, Kaseva ME. Critical review of industrial and medical waste practices in Dar es Salaam City. Resources, Conservation and Recycling. 1999 Mar 1;25(3-4):271-87.

[89] Shiferaw Y, Abebe T, Mihret A. Hepatitis B virus infection among medical waste handlers in Addis Ababa, Ethiopia. BMC research notes. 2011 Dec;4(1):1-7.

[90] Anagaw B, Shiferaw Y, Anagaw B, Belyhun Y, Erku W, Biadgelegn F, Moges B, Alemu A, Moges F, Mulu A. Seroprevalence of hepatitis B and C viruses among medical waste handlers at Gondar town Health institutions, Northwest Ethiopia. BMC research notes. 2012 Dec;5(1):1-0.

[91] Akele ES, Tarekegn MM. Assessment of dioxin and furan emission levels and management practices in Addis Ababa, Ethiopia. Journal of Health and Pollution. 2017 Sep;7(15):85-94.

[92] Benson NU, Adedapo AE, Fred-Ahmadu OH, Williams AB, Udosen ED, Ayejuyo OO, Olajire AA. New ecological risk indices for evaluating heavy metals contamination in aquatic sediment: A case study of the Gulf of Guinea. Regional Studies in Marine Science. 2018 Feb 1;18:44-56.

[93] Okafor-Yarwood, Ifesinachi, and Ibukun Jacob Adewumi. Toxic waste dumping in the Global South as a form of environmental racism: Evidence from the Gulf of Guinea. African Studies 79.3 (2020): 285-304.

[94] Rawat M, Singh UK, Mishra AK, Subramanian V. Methane emission and heavy metals quantification from selected landfill areas in India. Environmental monitoring and assessment. 2008 Feb;137(1):67-74.

[95] Singh M, Thind PS, John S. Health risk assessment of the workers exposed to the heavy metals in e-waste recycling sites of Chandigarh and Ludhiana, Punjab, India. Chemosphere. 2018 Jul 1;203:426-33.

[96] Awasthi AK, Zeng X, Li J. Relationship between e-waste recycling and human health risk in India: a critical review. Environmental Science and Pollution Research. 2016 Jun;23(12):11509-32.

[97] Pradhan JK, Kumar S. Informal e-waste recycling: environmental risk assessment of heavy metal contamination in Mandoli industrial area, Delhi, India. Environmental Science and Pollution Research. 2014 Jul;21(13):7913-28.

[98] Wath SB, Dutt PS, Chakrabarti T. E-waste scenario in India, its management and implications. Environmental monitoring and assessment. 2011 Jan;172(1):249-62.

[99] Shivpuri KK, Lokeshappa B, Kulkarni DA, Dikshit AK. Metal leaching potential in coal fly ash. American Journal of Environmental Engineering. 2011;1(1):21-7.

[100] Sharma S, Goel A, Gupta D, Kumar A, Mishra A, Kundu S, Chatani S, Klimont Z. Emission inventory of non-methane volatile organic compounds from anthropogenic sources in India. Atmospheric Environment. 2015 Feb 1;102:209-19.

[101] Majumdar D, Srivastava A. Volatile organic compound emissions from municipal solid waste disposal sites: A case study of Mumbai, India. Journal of the Air & Waste Management Association. 2012 Apr 1;62(4):398-407.

[102] Hoque RR, Khillare PS, Agarwal T, Shridhar V, Balachandran S. Spatial and temporal variation of BTEX in the urban atmosphere of Delhi, India. Science of the total environment. 2008 Mar 15;392(1):30-40.

[103] Kumar A, Singh D, Kumar K, Singh BB, Jain VK. Distribution of VOCs in urban and rural atmospheres of subtropical India: temporal variation, source attribution, ratios, OFP and risk assessment. Science of the Total Environment. 2018 Feb 1;613:492-501.

[104] Babu BR, Parande AK, Rajalakshmi R, Suriyakala P, Volga M. Management of biomedical waste in India and other countries: a review. Journal of International Environmental Application & Science. 2009 Jan;4(1):65-78.

[105] Solberg KE. Trade in medical waste causes deaths in India. The Lancet. 2009 Mar 28;373(9669):1067.

[106] Wiedinmyer, Christine, Robert J. Yokelson, and Brian K. Gullett. Global emissions of trace gases, particulate matter, and hazardous air pollutants from open burning of domestic waste. Environmental science & technology 48.16 (2014): 9523-9530.

[107] Masoabi, T. G. Understanding public knowledge and awareness of e-waste management practices in Maseru, Lesotho. Diss. North-West University (South Africa), 2020.

[108] Gwimbi, Patrick, and Masepele Jenette Selimo. Heavy metal concentrations in sediments and Cyprinus carpio from Maqalika Reservoir–Maseru, Lesotho: An analysis of potential health risks to Fish consumers. Toxicology reports 7 (2020): 475-479.

[109] David, Victor Emery, Yasinta John, and Shahid Hussain. Rethinking sustainability: a review of Liberia's municipal solid waste management systems, status, and challenges. Journal of Material Cycles and Waste Management (2020): 1-19.

[110] David Jr, V. Emery, et al. Health Care Waste Management Practices in Liberia: An Investigative Case Study. International Journal of Waste Resources (2016).

[111] Cholez, Céline, and Pascale Trompette. A mundane infrastructure of energy poverty: The informal trading of second-hand car batteries in Madagascar. Journal of Material Culture 25.3 (2020): 259-288.

[112] Elmamy CA, Abdellahi BM, Er-Raioui H, Dartige A, Zamel ML, Deida PM. Hydrocarbon pollution in Atlantic coast of Mauritania (Levrier Bay Zone): Call for sustainable management. Marine Pollution Bulletin. 2021 May 1;166:112040.

[113] Vallaeys T, Klink SP, Fleouter E, Le Moing B, Lignot JH, Smith AJ. Bioindicators of marine contaminations at the frontier of environmental monitoring and environmental genomics. pharmaceuticals. 2017;57:58.

[114] Gioia R, Eckhardt S, Breivik K, Jaward FM, Prieto A, Nizzetto L, Jones KC. Evidence for major emissions of PCBs in the West African region. Environmental science & technology. 2011 Feb 15;45(4):1349-55.

[115] Cerón Bretón, Julia Griselda, et al. Health risk assessment of the levels of BTEX in ambient air of one urban site located in Leon, Guanajuato, Mexico during two climatic seasons. Atmosphere 11.2 (2020): 165.

[116] Flores-Ramírez, Rogelio, et al. Exposure to mixtures of pollutants in Mexican children from marginalized urban areas. Annals of global health 84.2 (2018): 250.

[117] Carballo-Pat, C. G., et al. Levels of BTEX and criteria pollutants in ambient air of San Nicolas de los Garza, Nuevo Leon, Mexico during summer 2013. Latest trends in Energy, Environment and Development, Proceedings of the 7th International Conference on Environmental and Geological Sciences and Engineering (EG'14).

[118] Gullett, Brian K., et al. PCDD/F, PBDD/F, and PBDE emissions from open burning of a residential waste dump. Environmental science & technology 44.1 (2010): 394-399.

[119] Cruz-Sotelo, Samantha E., et al. E-waste supply chain in Mexico: Challenges and opportunities for sustainable management. Sustainability 9.4 (2017): 503.

[120] Saldaña-Durán, Claudia E., et al. Environmental pollution of E-waste: Generation, collection, legislation, and recycling practices in Mexico. Handbook of Electronic Waste Management. Butterworth-Heinemann, 2020. 421-442.

[121] Piazza, Rossano, et al. PCBs and PAHs in surficial sediments from aquatic environments of Mexico City and the coastal states of Sonora, Sinaloa, Oaxaca and Veracruz (Mexico). Environmental geology 54.7 (2008): 1537-1545.

[122] Zhang, Tingting, et al. Emissions of unintentional persistent organic pollutants from open burning of municipal solid waste from developing countries. Chemosphere 84.7 (2011): 994-1001.

[123] Croitoru, Lelia, and Maria Sarraf. How Much Does Environmental Degradation Cost? The Case of Morocco. Journal of Environmental Protection 9.3 (2018): 254-265.

[124] Baali, Ayoub, et al. Bile metabolites of polycyclic aromatic hydrocarbons (PAHs) in three species of fish from Morocco. Environmental Sciences Europe 28.1 (2016): 1-6.

[125] Pavoni, B., et al. Environmental pollutants and organic carbon content in sediments from an area of the Moroccan Mediterranean coast. Toxicological and Environmental Chemistry 84.1 (2003): 53-67.

[126] Zouir, Abdelali, et al. Use of semipermeable membrane devices for assessment of air quality in Tangier (Morocco). International Journal of Environmental and Analytical Chemistry 89.8-12 (2009): 917-928.

[127] Dahchour, Abdelmalek, and Souad El Hajjaji. Management of solid waste in Morocco. Waste Management in MENA Regions. Springer, Cham, 2020. 13-33.

[128] Kamau JN, Kuschk P, Machiwa J, Macia A, Mothes S, Mwangi S, Munga D, Kappelmeyer U. Investigating the distribution and fate of Al, Cd, Cr, Cu, Mn, Ni, Pb and Zn in sewage-impacted mangrove-fringed creeks of Kenya, Tanzania and Mozambique. Journal of soils and sediments. 2015 Dec;15(12):2453-65.

[129] Ricolfi L, Barbieri M, Muteto PV, Nigro A, Sappa G, Vitale S. Potential toxic elements in groundwater and their health risk assessment in drinking water of Limpopo National Park, Gaza Province, Southern Mozambique. Environmental geochemistry and health. 2020 Jan 6:1-3.

[130] Verlicchi P, Grillini V. Surface water and groundwater quality in South Africa and mozambique—Analysis of the Most critical pollutants for drinking purposes and challenges in water treatment selection. Water. 2020 Jan;12(1):305.

[131] Ajah KC, Ademiluyi J, Nnaji CC. Spatiality, seasonality and ecological risks of heavy metals in the vicinity of a degenerate municipal central dumpsite in Enugu, Nigeria. Journal of Environmental Health Science and Engineering. 2015 Dec;13(1):1-5.

[132] Manhart A, Osibanjo O, Aderinto A, Prakash S. Informal e-waste management in Lagos, Nigeria–socio-economic impacts and feasibility of international recycling co-operations. Final report of component. 2011;3:1-29.

[133] Jiang, Bo, et al. Impacts of heavy metals and soil properties at a Nigerian e-waste site on soil microbial community. Journal of hazardous materials 362 (2019): 187-195.

[134] Isimekhai, Khadijah A., et al. Heavy metals distribution and risk assessment in soil from an informal E-waste recycling site in Lagos State, Nigeria. Environmental Science and Pollution Research 24.20 (2017): 17206-17219.

[135] Adeyi, Adebola Abosede, and Peter Oyeleke. Heavy metals and polycyclic aromatic hydrocarbons in soil from e-waste dumpsites in Lagos and Ibadan, Nigeria. Journal of Health and Pollution 7.15 (2017): 71-84.

[136] Manhart, Andreas, et al. Informal e-waste management in Lagos, Nigeria–socio-economic impacts and feasibility of international recycling co-operations. Final report of component 3 (2011): 1-129.

[137] Ololade, Isaac Ayodele, et al. Concentrations and toxic equivalency of polycyclic aromatic hydrocarbons (PAHs) and polychlorinated biphenyl (PCB) congeners in groundwater around waste dumpsites in South-West Nigeria. Archives of Environmental Contamination and Toxicology 80.1 (2021): 134-143.

[138] Nazeer S, Hashmi MZ, Malik RN. Heavy metals distribution, risk assessment and water quality characterization by water quality index of the River Soan, Pakistan. Ecological indicators. 2014 Aug 1;43:262-70.

[139] Tufail M, Khalid S. Heavy metal pollution from medical waste incineration at Islamabad and Rawalpindi, Pakistan. Microchemical Journal. 2008 Oct 1;90(1):77-81.

[140] Jiang L, Cheng Z, Zhang D, Song M, Wang Y, Luo C, Yin H, Li J, Zhang G. The influence of e-waste recycling on the molecular ecological network of soil microbial communities in Pakistan and China. Environmental Pollution. 2017 Dec 1;231:173-81.

[141] Iqbal M, Breivik K, Syed JH, Malik RN, Li J, Zhang G, Jones KC. Emerging issue of e-waste in Pakistan: a review of status, research needs and data gaps. Environmental Pollution. 2015 Dec 1;207:308-18.

[142] Zuberi MJ, Ali SF. Greenhouse effect reduction by recovering energy from waste landfills in Pakistan. Renewable and Sustainable Energy Reviews. 2015 Apr 1;44:117-31.

[143] Mansha M, Saleemi AR, Naqvi JH. Status and spatial visualization of toxic pollutants (BTEX) in urban atmosphere. Advances in Chemical Engineering and Science. 2011 Oct 26;1(4):231-8.

[144] Tufail M, Khalid S. Heavy metal pollution from medical waste incineration at Islamabad and Rawalpindi, Pakistan. Microchemical Journal. 2008 Oct 1;90(1):77-81.

[145] Ali M, Kuroiwa C. Status and challenges of hospital solid waste management: case studies from Thailand, Pakistan, and Mongolia. Journal of Material Cycles and Waste Management. 2009 Sep;11(3):251-7.

[146] Hamuna B, Tanjung RH. Heavy metal content and spatial distribution to determine the water pollution index in depapre waters, Papua, Indonesia. Current Applied Science and Technology 2021:1-1.

[147] Ba, A. Ndong, et al. Individual exposure level following indoor and outdoor air pollution exposure in Dakar (Senegal). Environmental Pollution 248 (2019): 397-407.

[148] Sidoumou, Z., et al. Heavy metal concentrations in molluscs from the Senegal coast. Environment international 32.3 (2006): 384-387.

[149] Diop, Cheikh, et al. Assessment of trace metals contamination level, bioavailability and toxicity in sediments from Dakar coast and Saint Louis estuary in Senegal, West Africa. Chemosphere 138 (2015): 980-987.

[150] Haefliger, Pascal, et al. Mass lead intoxication from informal used lead-acid battery recycling in Dakar, Senegal. Environmental health perspectives 117.10 (2009): 1535-1540.

[151] Kapepula, Ka-Mbayu, et al. A multiple criteria analysis for household solid waste management in the urban community of Dakar. Waste Management 27.11 (2007): 1690-1705.

[152] Dieng, Cheikh, et al. Biomedical waste management in Dakar, Senegal: legal framework, health and environment issues; policy and program options. Cities & Health (2020): 1-15.

[153] Fredericks, Rosalind. Disorderly Dakar: The cultural politics of household waste in Senegal's capital city. The Journal of Modern African Studies 51.3 (2013): 435-458.

[154] Thiam, Sokhna, et al. Prevalence of diarrhoea and risk factors among children under five years old in Mbour, Senegal: a cross-sectional study. Infectious diseases of poverty 6.1 (2017): 1-12.

[155] Mbengue, Ramatoulaye, Mame Demba Thiam, and Vieux Boukhaly Traore. Impacts of household solid urban waste on the coast of Ngor (Dakar/Senegal). European Journal of Earth and Environment Vol 2.1 (2015).

[156] Moeckel C, Breivik K, Nøst TH, Sankoh A, Jones KC, Sweetman A. Soil pollution at a major West African E-waste recycling site: Contamination pathways and implications for potential mitigation strategies. Environment international. 2020 Apr 1;137:105563.

[157] Mansaray AS, Senior AB, Samai IJ, Koroma BM. Exposure to Dioxins and Furans at the Bormeh Kingtom Dumpsite in the Western Area of Sierra Leone. Natural Resources. 2015;6(09):491.

[158] Sankoh FP, Yan X, Tran Q. Environmental and health impact of solid waste disposal in developing cities: a case study of granville brook dumpsite, Freetown, Sierra Leone. Journal of Environmental Protection. 2013 Jul 2;2013.

[159] Derflerová Brázdová Z, Pomerleau J, Fiala J, Vorlová L, Müllerová D. Heavy metals in hair samples: a pilot study of anaemic children in Kazakhstan, Kyrgyzstan and Uzbekistan. Central European journal of public health. 2014 Dec 1;22(4):273-6.

[160] Kodirov O, Shukurov N. Heavy metal distribution in soils near the Almalyk mining and smelting industrial area, Uzbekistan. Acta Geologica Sinica-English Edition. 2009 Oct;83(5):985-90.

[161] Bandowe BA, Shukurov N, Kersten M, Wilcke W. Polycyclic aromatic hydrocarbons (PAHs) and their oxygen-containing derivatives (OPAHs) in soils from the Angren industrial area, Uzbekistan. Environmental pollution. 2010 Sep 1;158(9):2888-99.
